# Plant Carbohydrate Scavenging through TonB-Dependent Receptors: A Feature Shared by Phytopathogenic and Aquatic Bacteria

**DOI:** 10.1371/journal.pone.0000224

**Published:** 2007-02-21

**Authors:** Servane Blanvillain, Damien Meyer, Alice Boulanger, Martine Lautier, Catherine Guynet, Nicolas Denancé, Jacques Vasse, Emmanuelle Lauber, Matthieu Arlat

**Affiliations:** 1 Laboratoire des Interactions Plantes-Microorganismes, Centre National de la Recherche Scientifique (CNRS)/Institut National de la Recherche Agronomique (INRA) UMR2594/441, Castanet-Tolosan, France; 2 Université Paul Sabatier, Toulouse III, Toulouse, France; University of British Columbia, Canada

## Abstract

TonB-dependent receptors (TBDRs) are outer membrane proteins mainly known for the active transport of iron siderophore complexes in Gram-negative bacteria. Analysis of the genome of the phytopathogenic bacterium *Xanthomonas campestris* pv. *campestris* (*Xcc*), predicts 72 TBDRs. Such an overrepresentation is common in *Xanthomonas* species but is limited to only a small number of bacteria. Here, we show that one *Xcc* TBDR transports sucrose with a very high affinity, suggesting that it might be a sucrose scavenger. This TBDR acts with an inner membrane transporter, an amylosucrase and a regulator to utilize sucrose, thus defining a new type of carbohydrate utilization locus, named CUT locus, involving a TBDR for the transport of substrate across the outer membrane. This sucrose CUT locus is required for full pathogenicity on *Arabidopsis*, showing its importance for the adaptation to host plants. A systematic analysis of *Xcc* TBDR genes and a genome context survey suggested that several *Xcc* TBDRs belong to other CUT loci involved in the utilization of various plant carbohydrates. Interestingly, several *Xcc* TBDRs and CUT loci are conserved in aquatic bacteria such as *Caulobacter crescentus, Colwellia psychrerythraea, Saccharophagus degradans, Shewanella* spp., *Sphingomonas* spp. or *Pseudoalteromonas* spp., which share the ability to degrade a wide variety of complex carbohydrates and display TBDR overrepresentation. We therefore propose that TBDR overrepresentation and the presence of CUT loci designate the ability to scavenge carbohydrates. Thus CUT loci, which seem to participate to the adaptation of phytopathogenic bacteria to their host plants, might also play a very important role in the biogeochemical cycling of plant-derived nutrients in marine environments. Moreover, the TBDRs and CUT loci identified in this study are clearly different from those characterized in the human gut symbiont *Bacteroides thetaiotaomicron*, which allow glycan foraging, suggesting a convergent evolution of TBDRs in Proteobacteria and Bacteroidetes.

## Introduction

Bacteria are able to colonize a wide variety of habitats, including the most extreme environments and even living organisms. This remarkable feature likely reflects a high degree of adaptability and the presence of specific genetic programs devoted to the exploitation of nutrients present in these diverse habitats. The bacterial ability to use defined carbohydrates to support cell survival or growth implies the availability of these substrates in their habitat. Moreover, it requires the recognition of these molecules and the coordinated induction of particular uptake systems and of metabolic enzymes [1], [2]. Characterization of the repertoire of genes involved in signal perception or transduction and in carbohydrate utilization, in conjunction with analysis of the regulation of their expression, is likely to provide key information about the interaction and adaptation of bacteria with their environment [3].

The analysis and comparison of complete genomic sequences of numerous bacteria from diverse phylogenies and habitats have shown a relationship between the ecological niches that a bacterium occupies and the proportion of genes involved in signal perception and transduction. Bacteria that inhabit stable environments (extremophiles, obligate parasites or symbionts), which generally have small genomes, possess fewer sensory and regulatory genes than free-living bacteria found in complex and changing environments such as those living in soil or in association with plants [2]. Therefore, it was proposed that sensors and regulators can be used as “descriptors of bacterial lifestyle” [2].

With the aim to study the molecular mechanisms controlling adaptation of phytopathogenic bacteria to their host-plants, we undertook a global analysis of receptors and regulators of *Xanthomonas campestris* pv*. campestris* (*Xcc*), the causal agent of black rot of crucifers. This pathogen infects a wide range of *Brassicaceae* plants of economic interest, including cabbage, cauliflower and radish as well as the model plant *Arabidopsis thaliana*. This epiphytic bacterium naturally infects host plants *via* wounds in the leaves or hydathodes which are specialized pores on the leaf margins of higher plants that connect to the vascular system. Then bacteria multiply and progress in vascular tissues [4], [5]. During the past two decades, classical molecular and genetic studies led to the characterization of several determinants controlling pathogenicity of *Xcc*, such as secretion of extracellular plant cell wall degrading enzymes [6]–[8], cell-cell signaling [9], [10], biofilm formation [11] and *hrp* genes coding for a type III secretion system [12], [13]. The characterization of new virulence factors should now be greatly facilitated by the availability of complete genome sequences of two *Xcc* strains (strain ATCC33913 [14] and strain 8004 [15]). Moreover, comparative genomics would improve the analysis of virulence and host adaptation in *Xanthomonas*, since the genomic sequences of four other *Xanthomonas* strains displaying different host specificities and representing three other species are also available, i.e. *Xanthomonas axonopodis* pv. *citri* (*Xac*), the causal agent of citrus canker [14], *Xanthomonas campestris* pv. *vesicatoria* (*Xcv*), the causative agent of bacterial spot disease on pepper and tomato plants [16], and *Xanthomonas oryzae* pv. *oryzae* (*Xoo*) (strain Kacc10331 [17] and strain MAFF311018 [18]), the causal agent of bacterial blight of rice. Thus, the *Xanthomonas* genus, which affects two major model plants (*Arabidopsis* and rice), constitutes a very attractive model to study plant-pathogen interactions.

Our analysis of the *Xcc* (ATCC33913) genome revealed an overrepresentation of a particular family of receptors, named TonB-dependent receptors (TBDRs). These proteins are located in the outer membrane of Gram-negative bacteria and are mainly known to transport iron-siderophore complexes and vitamin B12 into the periplasm [19]. In most cases, the expression of the genes encoding these receptors is under the control of the Fur (Ferric uptake regulator) repressor and activated under conditions of iron starvation [20]. In contrast to diffusion through porins, transport *via* TBDRs requires energy which is provided by the inner membrane energy-coupling TonB-ExbB-ExbD protein complex [21].

The exploration of complete genome sequences of 226 Gram negative bacteria showed that the overrepresentation of TBDRs is restricted to a small proportion of these bacteria, but is a common trait of all sequenced *Xanthomonas* species. Interestingly, most of the bacteria displaying this particularity have diverse lifestyles and belong to different taxonomical lineages, but they all share the ability to exploit complex carbohydrates. Therefore, we postulated that some *Xcc* TBDRs might be involved in the transport of plant-derived molecules. This hypothesis was recently reinforced with the characterization of a TBDR, named MalA, in the oligotrophic aquatic bacterium *Caulobacter crescentus*, which transports maltodextrins [22].

A systematic study of *Xcc* TBDRs, based on mutagenesis, expression analyses and transport assays identified one *Xcc* TBDR involved in the transport of sucrose, a major plant sugar. This TBDR gene is required for full virulence on *Arabidopsis* and is associated with genes required for sucrose metabolism, thus forming a “sucrose utilization locus”. Our study also suggests the existence of other TBDR-containing loci involved in the utilization of plant cell wall compounds which are conserved in a wide range of bacteria displaying TBDR overrepresentation.

## Results

### Identification of *Xcc* TBDRs

TBDR proteins consist of two domains, with a C-terminal membrane embedded β-barrel domain that is sealed by the N-terminal plug domain [23]–[27]. Hidden Markov models (HMMs), PF00593 and PF07715, corresponding to these two domains, are available in the Pfam database [28]. Seventy-six proteins carrying one or both domains were detected in the proteome of the *Xcc* ATCC33913 strain (Table S1). Among these proteins, 64 possess both domains. The remaining 12 proteins, which possess only one of the two domains, can be divided into three groups: 3 proteins (*XCC2208, XCC4131* and *XCC4132*) which do not seem to have a canonical plug domain, one protein (*XCC2497*) which has no domain detected by the PF00593 HMM, and finally 8 proteins (*XCC0304-0305, XCC1750-1751, XCC3215-3216* and *XCC3270-3271*) which are truncated and correspond to 4 pseudogenes having a frame-shift in their coding region (Table S1). Each of these 4 pseudogenes was thereafter studied as a unique entity.

Other conserved features of TBDRs were then used to further characterize these identified proteins. In the N-terminal part of the plug domain, TBDRs display a conserved sequence, the TonB-box, that interacts with the TonB protein [26], [27], [29]. A TonB-box, with the *Xcc* consensus sequence tLDXVXV (lower case indicates less highly conserved amino acid, X indicates any amino acid) was detected in all studied proteins (Table S1). The localization of TBDRs in the outer membrane implies their transport across the inner membrane and thus the presence of a signal peptide at their N-terminal end. The proteins were therefore analyzed for the presence of such a motif. TBDRs which were not predicted to possess a signal peptide in the annotation proposed by da Silva and colleagues [14] were manually re-annotated, thus revealing putative signal peptides (Table S1). Out of 72 proteins, 1 TBDR (XCC2385) does not have a signal peptide. Finally, the 12 C-terminal amino acids of outer membrane proteins (OMPs) form a membrane anchoring β-sheet with the last residue being aromatic (F in the vast majority of OMPs) [30], [31]. The 12 C-terminal amino acid of 55 of the proteins studied here are predicted to form a potential β-sheet, ending with an aromatic residue (Table S1). Among the TBDRs which did not display a typical C-terminal sequence, 8 have a β-sheet ending with an aromatic amino acid followed by a short extension of 1 to 4 amino acids (Table S1).

Taking all this information together, 48 proteins possess all typical features of TBDRs in *Xcc* ATCC33913 and were thus considered as putative functional TBDRs. The other proteins lacking or having degenerated TBDR domains were also included in our study and then named Ps-TBDRs (for Pseudo-TBDRs) (Table S1).

Finally, some TBDRs contain a N-terminal extension, located between the signal peptide and the plug domain [32]. Two types of N-terminal extension have been identified: the transducer and the Oar-like extensions [33]. In *Xcc*, among the 9 TBDRs having an N-terminal extension, one is a member of the TBDR-transducer subclass, seven are in the Oar-like subclass and one displays a N-terminal extension that does not correspond to the two types already identified (Table S1). Among these TBDRs, only two have all canonical features of TBDRs.

### TBDRs are overrepresented in *Xanthomonas* spp. and in bacteria scavenging complex carbohydrates

**Figure 1:**
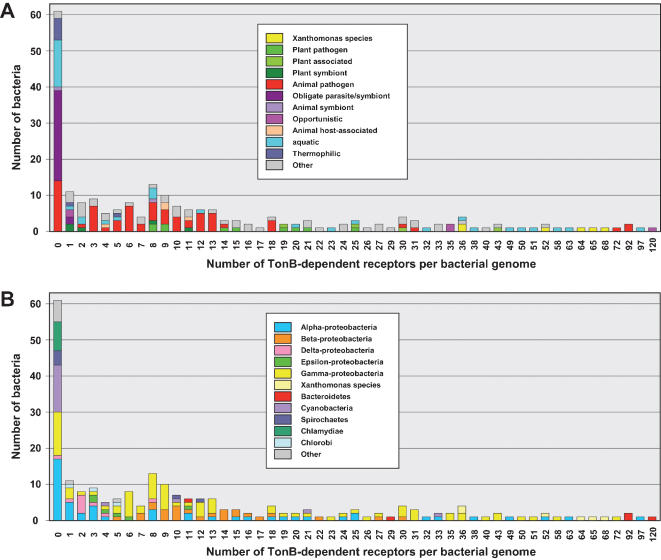
Occurrence of TonB-dependent receptor (TBDR) genes in 226 completely sequenced Gram negative bacterial genomes, in ecological (A) or phylogenetic (B) contexts. TBDRs were detected by screening the Pfam database and the UniprotKB protein knowledge database using the two Pfam HMMs (PF07715, Plug; PF00593, TonB_dep_Rec). Only proteins displaying the two Pfam domains were considered. The habitat of bacteria (A) and their phylogeny (B) are represented by colour codes (see the legend boxes).

A survey of TBDRs was performed in 226 eubacterial completely sequenced genomes, by identifying proteins detected by both PF07715 and PF00593 HMMs. This analysis showed that most bacteria (71%) hold less than 14 TBDRs per proteome, whereas the remaining bacteria have a very broad variation in TBDR number, ranging from 14 to 120 (Figure 1 and Table S2). In fact, 27% of the bacteria studied here have no TBDR proteins and 43% possess between 1 and 13 TBDRs. Only a very small proportion of bacteria (15.5%) possesses more than 30 TBDRs, thus forming a particular class in which TBDRs seem to be over represented. It is worth noting that most bacteria in this class either belong to the α or γ-Proteobacteria classes or to the *Bacteroides* genus (Figure 1 and Table S2). The ratios between the number of TBDRs found in each proteome and the genome size or the number of annotated coding sequences (CDS) displayed a very similar distribution pattern (Table S2), thus showing that there was no major bias due to annotation. Therefore, the TBDR/Mbp ratio was used to define several bacterial classes: bacteria displaying a ratio higher than 5 were considered as members of a class showing TBDR overrepresentation, while those having a ratio ranging from 3 to 5 belong to an intermediary class. The 4 *Xanthomonas* species whose genomes have been sequenced belong to the class displaying TBDR overrepresentation (Table S2). It is worth noting that phytopathogenic bacteria, such as *Pseudomonas syringae* pathovars or *Erwinia carotovora* subsp. *Atroseptica* belong to the intermediary class.

### Seven *Xcc* TBDR and two Ps-TBDR genes are Fur-regulated

As TBDRs are mainly known to be involved in iron uptake, it was important to determine how many of these receptors are assigned to this function in *Xcc*. In most cases, the expression of genes encoding TBDRs involved in iron transport is regulated by the iron status in the medium. These genes are activated under iron depletion conditions and repressed under iron repletion conditions *via* the Fur repressor (Ferric-uptake regulator), that binds to a specific DNA sequence element called the “Fur-box”, which is found in the target promoters of iron-regulated genes [20], [34], [35]. These features led us to analyze the regulation by the iron status for all identified *Xcc* TBDR and Ps-TBDR genes.

**Figure 2:**
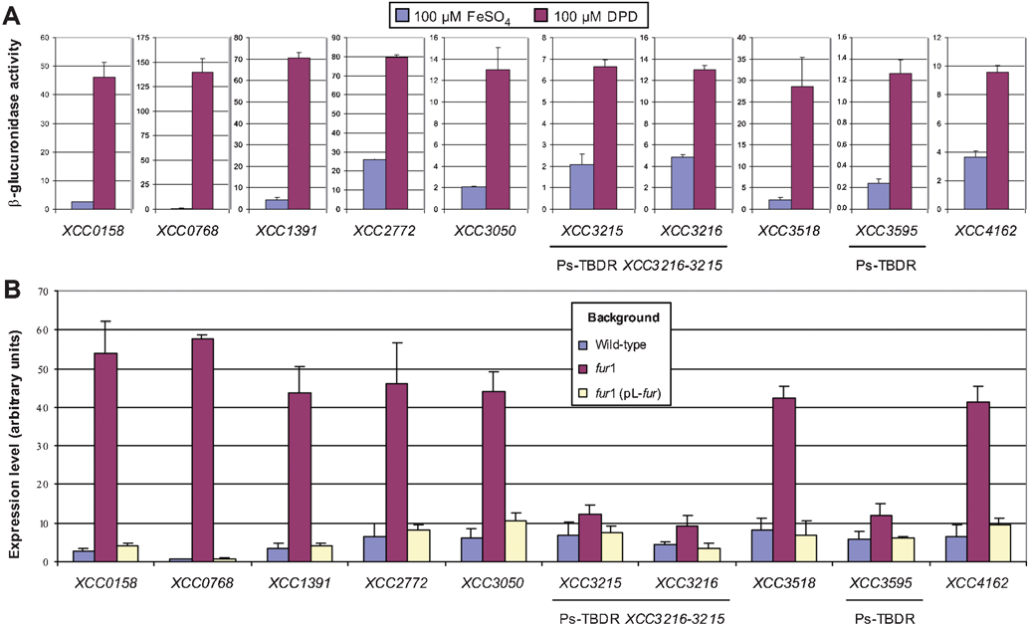
Expression levels of 9 *Xanthomonas campestris* pv. *campestris* TonB-dependent receptor genes. (A) β-glucuronidase assays, performed in at least two independent experiments with pVO155 insertion mutant strains, cultivated in minimal medium supplemented with iron (100 µM FeSO_4_) or containing an iron chelator (100 µM 2,2′-dipyridyl; DPD). (B) Real-time quantitative RT-PCR (qRT-PCR), performed in at least three independent experiments, on RNA extracted from the wild-type strain, a *fur* mutant strain (*fur*1) or from a complemented strain [*fur*1 (pL-*fur*)], cultivated in minimal medium containing 100 µM FeSO_4_. Calculation of relative expression includes normalization against the endogenous control gene 16S RNA.

The 76 *Xcc* TBDR and Ps-TBDR genes were mutated by insertion of the suicide plasmid pVO155 [36], leading to transcriptional fusion with the promoterless *uidA* reporter gene. Two insertions (in *XCC1990* and *XCC3209*) were found unstable. Thus, we constructed deletion-mutations in these genes using the *cre-lox* system [37], [38]. In order to analyze the expression of these 2 deleted TBDR genes, their promoter regions were cloned upstream of a promoterless *lacZ* gene in a reporter plasmid (see Materials and Methods), and these constructions were introduced into the wild-type strain. Using β-glucuronidase or β-galactosidase expression assays, we monitored the expression of all *Xcc* TBDRs and Ps-TBDRs in iron-repleted and -depleted media. Seven genes (*XCC0158, XCC0768, XCC1391, XCC2772, XCC3050, XCC3518*, and *XCC4162*) and 2 Ps-TBDR genes (*XCC3216*-*3215 and XCC3595*) are induced by iron starvation, compared to iron-replete conditions (Figure 2A). With the exception of Ps-TBDR *XCC3216-3215*, this regulation pattern was confirmed by quantitative real-time reverse transcriptase polymerase chain reaction (qRT-PCR) in a wild-type background (data not shown). We then checked whether these genes are under the control of the Fur regulator. XCC1470, the orthologue of the Fur regulator identified in *Xoo* [39] and in *Xanthomonas campestris* pv. *phaseoli* (*Xap*) [40], was mutated using the manganese method (see Materials and Methods). A point mutant in this latter gene was obtained (*fur*1). As expected for a *fur* mutation, this mutant produced more siderophores than the wild-type strain and this production remained unaffected in response to increased iron levels (from 1 to 50 µM), as determined by using the chrome azurol S assay (data not shown). It is worth noting that the *fur* mutant is unable to induce any disease symptoms (data not shown), as already reported in *Xoo* [39].

We then compared by qRT-PCR analysis the relative expression of the TBDR and Ps-TBDR genes induced by iron starvation, in the wild-type strain *versus* a *fur* mutant, in an iron-containing medium. As presented in Figure 2B, the expression of all these genes was repressed by the Fur protein. However, the repression level of the 2 Ps-TBDR genes was low (less than twofold). We confirmed that the deregulation phenotype of the *fur* mutant was the result of mutation in the *fur* gene by complementation experiments: the repression by iron of the 7 TBDR and the 2 Ps-TBDR genes was restored by providing the wild-type Fur protein on the pL-*XCC1470* plasmid (see Materials and Methods) in the *fur* mutant (Figure 2B).

### The 9 Fur-regulated TBDR/Ps-TBDR genes, but not the other *Xcc* TBDR genes, possess a putative Fur-box

**Figure 3:**
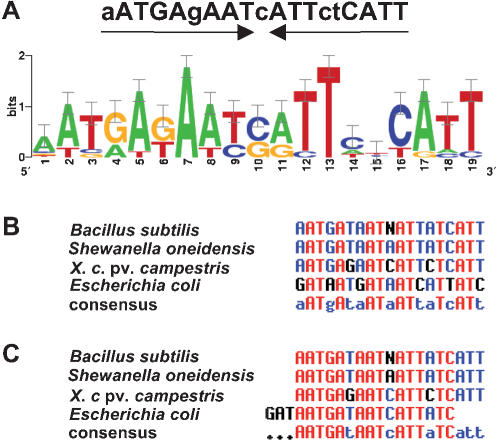
Palindromic consensus Fur-box sequence identified upstream of 9 *Xanthomonas campestris* pv. *campestris* Fur regulated TonB-dependent receptor genes. (A) Sequence logos generated by WebLogo (http://weblogo.berkeley.edu/, [123]) of the *Xcc* Fur-box motif as predicted by the MotifSampler program [41], [42]. (B and C) Multiple alignement generated with Multalin program [116] with previously proposed Fur-box consensus sequences of *Bacillus subtilis* [46], *Shewanella oneidensis* [45], *X. campestris* pv. *campestris* and *Escherichia coli* [44] (B) and after a 3 bp shift of the *E. coli* sequence (C).

The DNA regions located upstream of the 9 Fur-repressed TBDR and Ps-TBDR genes were analyzed using the MotifSampler program [41], [42] to identify putative Fur-boxes. For genes arranged in putative operons (*XCC3050* and *XCC0768*), the DNA region located upstream of the first gene of the putative operon was also studied. *XCC3595*, which belongs to the TBDR transducer subclass, is preceded by two genes displaying significant similarities with the *fecI* (*XCC3593*) and *fecR* (*XCC3594*) genes, coding for a sigma factor of the ECF subfamily and its anti-sigma factor, respectively. In most cases, the FecI/FecR system is associated with a TBDR of the transducer subclass and is involved in iron signaling and transport by regulating the expression of the TBDR gene and other “iron-associated” genes (for review see [33], [43]). We thus also analyzed the DNA regions upstream of these two genes. This analysis allowed the identification of a single significant 19-bp palindromic motif (9-1-9 inverted repeat) in the upstream region of most genes or operons studied here (Figure 3A, Table 1). For *XCC3595*, the palindromic motif was detected in the upstream region of *XCC3593* (*fecI*). For *XCC0768*, two motifs were identified: one in the upstream region of *XCC0767* and one in the coding region of this gene (Table 1). This motif (AATGAgAATcATTctCATT; lower case indicates less highly conserved) only showed a weak similarity with the *E. coli* 19 bp Fur-box consensus sequence (GATAATGATAATCATTATC) [44]: it matched this sequence in 10 bp positions out of 19 (52% identity). However, the conservation was significantly higher with Fur-box consensus sequences identified in *Shewanella oneidensis* (AATGATAATAATTATCATT; 84% identity) [45] and *Bacillus subtilis* (aaTGAtAATnATTaTCAtt; 84% identity) [46]. Interestingly, a multiple alignment of these 4 Fur-box consensus sequences showed that although the *E. coli* consensus sequence seems more divergent, it perfectly matches the 3 other sequences after a 3 bp shift (Figure 3B and C). These results suggest that the palindromic motif identified in this study might correspond to a possible *Xcc* Fur-box. We then explored DNA sequences located upstream of the other *Xcc* TBDR or Ps-TBDR genes as well as the entire *Xcc* genome sequence to detect putative Fur-boxes, using the MotifScanner program [42] or the PatScan pattern matcher software [47]. No putative Fur-box was detected in the promoter region of any other TBDR gene. These analyses identified more than 20 genes having candidate Fur-boxes in the region from −300 to +20 relative to the start of translation (data not shown). Several of these genes are orthologs or homologs of genes regulated by Fur in other bacteria, such as *feoA* (*XCC1834*) [48] and *bfd* (*XCC0481*) [49], or involved in iron storage such as *piuB* (*XCC3955*) [50], thus reinforcing the validity of our Fur-box consensus sequence. Altogether, this analysis suggests that only 7 TBDR and 2 Ps-TBDR are directly associated with iron uptake in *Xcc*. The role of the other TBDRs remained to be deciphered.

**Table 1:** Candidate Fur-boxes identified in the promoter regions of *Xanthomonas campestris* pv. *campestris* Fur regulated TonB-dependent receptor (TBDR) genes, TBDR containing operons and the XCC3593 FecI sigma factor gene.

Gene ID a	Name a	Putative function a	Putative Fur box sequences b	No. of matches/total	Distance from the start codon (bp) c
*XCC0158*	fpvA	ferripyoverdine receptor	AACGATAACGATTTACATT	14/19	136
*XCC0767*		conserved hypothetical protein	AATGAGAATGGCTCTTGAT	13/19	7
*XCC0768*	phuR	outer membrane hemin receptor	AATGAGAATGGTTATTATT	15/19	374
*XCC1391*	fhuA	iron receptor	TTTGATAACCATTCCCATT	14/19	9
*XCC2772*	fhuA	TonB-dependent receptor	AAGAAGAATGATTTGCATT	14/19	244
*XCC3049*	mphE	4-hydroxy-2-oxovalerate aldolase	CTTGATAATCATTCCCATC	14/19	41
*XCC3216*		outer membrane receptor	AATGTGAATCATTCCCATT	17/19	102
*XCC3518*	fpvA	ferripyoverdine receptor	AATAATATTCATTCTCACT	15/19	53
*XCC3593*	fecI	RNA polymerase sigma factor	AATGAGATCCATTCTCATT	17/19	48
*XCC4162*		ferrichrome-iron receptor 3	GATAAGAATCGTTATCATT	15/19	12

a Gene identification (ID), name and putative function from *Xanthomonas campestris* pv. *campestris* strain ATCC33913 as previously reported [14].

b Matches to the Fur consensus sequence AATGAgAATcATTctCATT, determined in this study, appear in boldface.

c Distance after start codon reannotation (see Table S1).

### Two TBDR genes belong to the *hrp* regulon of *Xcc*

**Figure 4:**
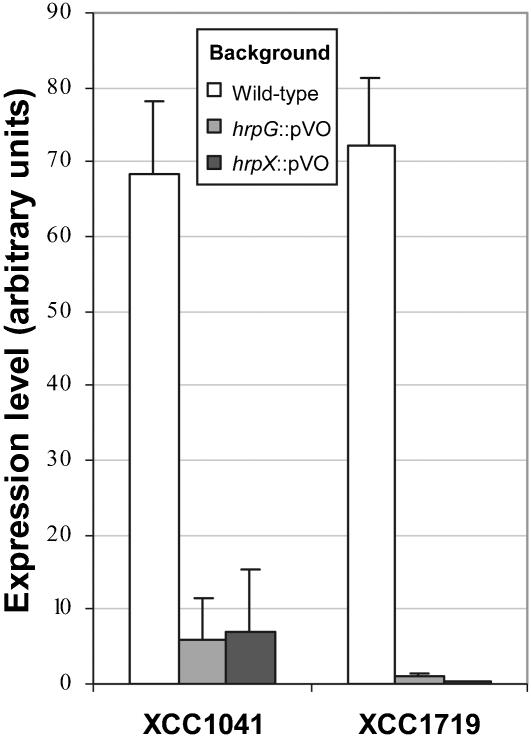
Regulation of *Xanthomonas campestris* pv. *campestris* TonB-dependent receptor genes *XCC1041* and *XCC1719* by *hrp* regulators. The relative expression was analyzed by real-time quantitaive RT-PCR (qRT-PCR), perfomed on RNA extracted from the wild-type strain and *hrpG* or *hrpX* insertion mutants (strains XP082 and XP083, respectively). Experiments were repeated at least three times. Calculation of relative expression includes normalization against the endogenous control gene 16S RNA.

During the study of TBDR gene promoters, we identified a pip/hrp_II_ box in the promoter of 2 TBDR genes: *XCC1041* and *XCC1719*. In *Xanthomonas, hrp* genes coding for a TTSS, as well as other functions related to pathogenicity are positively regulated by *hrpG/hrpX* regulatory genes [51]. HrpG regulates the expression of *hrpX*, which controls the expression of genes possessing a pip-box (TTCGCN_15_TTCGC) or a hrp_II_-box (TTCGN_16_TTCG) [52], [53]. By qRT-PCR experiments, we showed that *XCC1041* and *XCC1719* TBDR genes are activated by HrpG and HrpX (Figure 4). Interestingly, a mutant in the *XCC1719* TBDR gene is weakly affected in pathogenicity (data not shown, see below). This gene seems to be specific to *Xcc* as it is absent in the genomes of *Xcv, Xac, Xoo* and *Xf*, although its surrounding genes are conserved in syntenic regions in all these strains (Table S3). On the other hand, *XCC1041* is present in all *Xanthomonas* species (but absent in *Xf*) and encodes a TBDR with an atypical N-terminal extension.

### *Xcc* TBDRs and plant interactions: *XCC3358* plays a major role in pathogenicity

**Figure 5:**
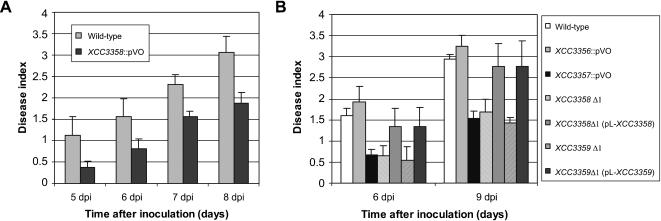
Quantitative analysis of the interaction between *Xanthomonas campestris* pv. *campestris* (*Xcc*) and *Arabidopsis thaliana Sf*-2 plants. (A) Pathogenicity tests with the *Xcc* wild-type strain and the *XCC3358* insertion mutant (*XCC3358*::pVO). (B) Pathogenicity and complementation tests with the *Xcc* wild-type strain, the *XCC3356* and *XCC3357* insertion mutants *(XCC3356*::pVO and *XCC3357*::pVO, respectively), the *XCC3358* and *XCC3359* deleted mutants (*XCC3358*D1 and *XCC3359*D1, respectively) and their corresponding complemented strains [*XCC3358*D1 (pL-*XCC3358*) and *XCC3359*D1 (pL-*XCC3359*), respectively]. Disease symptoms were scored 5 to 8 days after inoculation. Each inoculated leaf was individually scored as: no symptom = 0; weak chlorosis surrounding the wound sites = 1; strong V-shaped chlorosis = 2; developing necrosis = 3; leaf death = 4. The represented average disease scores and the standard deviations were calculated from the values of four plants with four inoculated leaves per plant.

**Figure 6:**
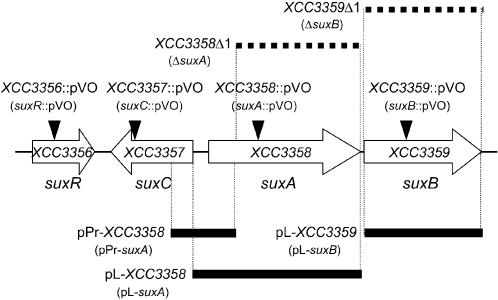
Genetic organization of the *Xanthomonas campestris* pv. *campestris sux* locus. Location of mutations are indicated above the map: arrowheads indicate pVO155 insertions and deleted sequences are represented by horizontal dotted bars. Regions cloned in plasmids are indicated below the map: horizontal thick black bars indicate sequences used for plasmid constructions.

In order to assess whether *Xcc* TBDRs control the interaction with plants, we tested the 76 TBDR mutants constructed in this study for pathogenicity on the *Arabidopsis thaliana Sf-2* ecotype. Among these mutants, 17 were altered in symptom development (data not shown). However, the alteration was weak and was not reproducible in all the tests we performed, suggesting either that their involvement is not crucial or that functional redundancy mask their individual contribution. However, the *XCC3358* insertion mutant was clearly and reproducibly altered in pathogenicity, showing a clear delay in symptom development in comparison to the wild-type strain (Figure 5A). The *XCC3358* gene is likely to form an operon with the downstream gene, *XCC3359* (Figure 6). The ortholog of the *XCC3359* gene, named *suh*, has been characterized in *Xanthomonas axonopodis* pv. *glycines* (*Xag*), the causal agent of bacterial pustule disease on soybean. This gene codes for a sucrose hydrolase, SUH, which plays a major role in sucrose metabolism in *Xag*. A mutant in this gene is moderately affected in pathogenicity on soybean [54]. This prompted us to construct a non polar deletion-mutation in *XCC3358* (*XCC3358*Δ1, see Materials and Methods and Figure 6). A deletion-mutant was also generated into *XCC3359* (*XCC3359*Δ1, see Materials and Methods and Figure 6). These two mutants showed an altered phenotype, i.e. delayed symptom development, similar to that observed with the pVO155 insertion mutant in *XCC3358* (Figure 5B). The non polar mutation in *XCC3358* was complemented by introducing the plasmid pL-*XCC3358*, which carries a functional *XCC3358* gene, into *XCC3358*Δ1 mutant (Figure 5B). This result confirms that the *XCC3358* TBDR plays a role in virulence. Similarly, the deleted mutant in *XCC3359* was complemented by a plasmid carrying this gene (Figure 5B).

### *XCC3358* TBDR belongs to a locus required for full pathogenicity

In *Xcc* genome, *XCC3358* and *XCC3359* are preceded by two other genes that might be related to sugar metabolism: *XCC3356* codes for a putative transcriptional repressor of the LacI/GalR-family and *XCC3357* encodes a putative sugar transporter of the major facilitator family, allowing the transport of substrate molecules through the inner membrane. We constructed insertion mutants in these two genes, presuming that they are monocistronic (Figure 6). These insertions lead to transcriptional fusions with the promoterless *uidA* reporter gene. The insertion mutant in *XCC3357* showed an altered phenotype on the *A. thaliana* Sf-2 ecotype, resembling that using *XCC3358* or *XCC3359* mutants, whereas the insertion mutant in *XCC3356* was not affected in pathogenicity (Figure 5B). These results suggest that *XCC3357*, *XCC3358* and *XCC3359* are required in the same process related to sucrose metabolism and/or transport. The phenotype of a mutant in *XCC3356* was not surprising since this gene encodes a putative repressor, which might negatively control the expression of the other genes of the locus. A mutation in this gene should induce the constitutive expression of the other genes without affecting their function (see below).

### The *XCC3356*-*3359* locus is required for sucrose utilization

**Figure 7:**
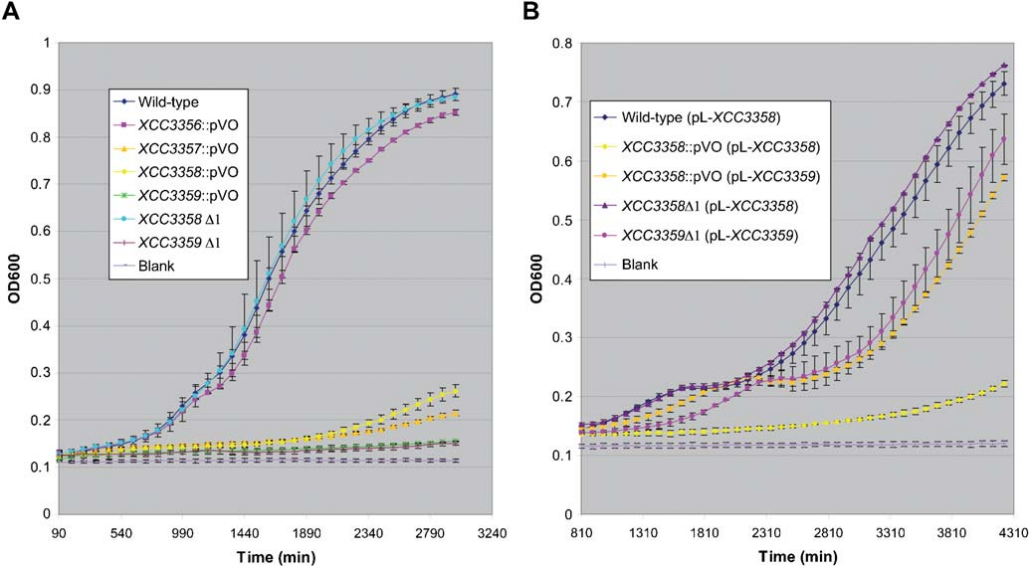
Growth of *Xanthomonas campestris* pv. *campestris* wild-type strain and mutants in minimal medium containing 20 mM sucrose. (A) Bacterial growth of insertion mutants *XCC3356*::pVO to *XCC3359*::pVO or deletion mutants *XCC3358*Δ1 and *XCC3359*Δ1, compared to the wild-type strain. (B) Growth of strains carrying plamids pL-*XCC3358* or pL-*XCC3359* allowing the constitutive expression of *XCC3358* and *XCC3359* genes, respectively. Bars represent standard deviations from 3 independent experiments.

Insertion mutants in *XCC3357, XCC3358* and *XCC3359* and the deletion mutant *XCC3359*Δ1 grew like the wild-type strain on minimal medium containing glucose or fructose (data not shown), but were all affected in growth on sucrose (20 mM) (Figure 7A). The growth of the insertion mutant in *XCC3356* and the *XCC3358*Δ1 mutant was not impaired in minimal medium containing sucrose. The fact that the insertion mutant in *XCC3358* was impaired in growth on sucrose whereas the deletion mutant in the same gene was not, suggested that the phenotype of the insertion mutant was due to a polar effect of the insertion on *XCC3359* gene expression. This hypothesis was confirmed by complementation experiments. The growth of insertion mutants in *XCC3358* and *XCC3359* on sucrose media was restored when XCC3359 was supplied *in trans* on the expression plasmid pL-*XCC3359*, which allows constitutive expression of the *XCC3359* gene. No complementation was observed when the *XCC3358* gene was supplied *in trans* in the *XCC3358* insertion mutant (Figure 7B). These results confirmed that *XCC3358* and *XCC3359* form an operon. They also pointed out that this putative operon and the entire locus is important for sucrose utilization in *Xcc*. Thus, we renamed this locus *sux*, for sucrose utilization in *Xanthomonas*. For the purpose of this study, the TBDR-amylosucrase operon was renamed *suxAB*, the putative sugar transporter gene, *suxC*, and the putative regulatory gene, *suxR* (see Figure 6). However, the role of the TBDR SuxA in sucrose utilization remained elusive: this transporter gene was not required for growth on sucrose, but a non polar mutant in this gene was altered in pathogenicity, like *suxB* and *suxC* insertion mutants. This observation suggested that this *suxA* gene might have a particular function.

### SuxA and SuxC allow sucrose uptake

To clarify the role of the SuxA TBDR, we investigated its involvement in sucrose transport, by performing sucrose uptake experiments using [^14^C]sucrose.

**Figure 8:**
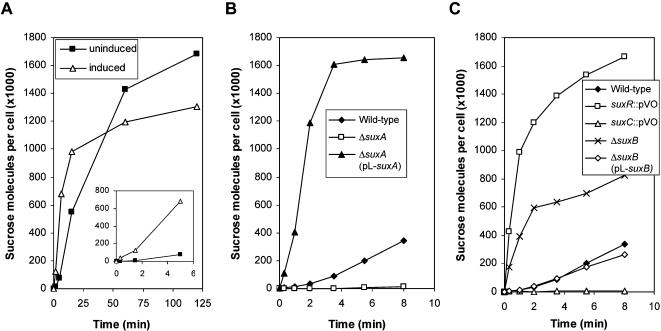
[^14^C]sucrose transport into *Xanthomonas campestris* pv. *campestris* (*Xcc*). (A) Transport of [^14^C]sucrose over 120 minutes into the *Xcc* wild-type strain precultured in minimal medium without sugar (uninduced) or supplemented with 20 mM sucrose (induced). (B and C) Transport of [^14^C]sucrose over 8 minutes into the *Xcc* wild-type strain, the insertion mutants in *suxR* and in *suxC* (*suxR*::pVO and *suxC*::pVO, respectively), the deleted mutants in *suxA* and *suxB* (Δ*suxA* and Δ*suxB*, respectively) and their corresponding complemented strains [Δ*suxA* (pL-*suxA*) and Δ*suxB* (pL-*suxB*), respectively]. Cells were grown in minimal medium without sucrose. Transport was measured using 0.5 µM [^14^C]sucrose.

First, the uptake rates into the *Xcc* wild-type strain were compared after an overnight preculture in the presence (induced) or absence (uninduced) of sucrose. As shown in figure 8A, induced cells took-up [^14^C]sucrose quicker than uninduced cells, but the values obtained after 2 hours of incubation were lower for induced cells. For further experiments, we worked with uninduced cells, in order to study the effect of different mutations on the transport induction.

Competition experiments were performed to test transport specificity. A 10-fold excess of unlabelled sucrose reduced the transport rate to 52% of the level obtained without unlabelled sucrose, and with a 100-fold or 200-fold excess, the transport rates were reduced to 10.5 and 7% respectively (Table 2). Moreover, the addition of unlabelled fructose or glucose (sucrose degradation sub-products) slightly affected sucrose transport; a 200-fold excess of these carbon sources reduced the sucrose transport to about 80% of the control level (Table 2).

**Table 2:** [^14^C]sucrose transport rates related to uninduced *Xanthomonas campestris* pv. *campestris* wild-type strain (*Xcc*568).

*Xcc* strains a	Genotype	Transport experiment conditions b	Time (min) c	% Transport
*Xcc*568		–	5	100.0
*Xcc*568 d		CCCP 20 µM	5	1.1
*Xcc*568		–	8	100.0
XP084	*suxR*::pVO	–	8	488.0
XP085	*suxC*::pVO	–	8	2.1
XP087	Δ*suxA*	–	8	3.5
XP088	Δ*suxB*	–	8	241.3
XP091	Δ*suxA* (pL-*suxA*)	–	8	484.3
XP094	ΔsuxB (pL-*suxB*)	–	8	76.4
XP100	*tonB; XCC0008*::pVO155	–	8	139.1
XP101	*tonB2; XCC1592*::pVO155	–	8	99.0
XP102	*tonB3; XCC1593*::pVO155	–	8	89.6
XP103	*tonB4; XCC2081*::pVO155	–	8	94.4
XP104	*tonB5; XCC2612*::pVO155	–	8	90.6
XP105	*tonB6; XCC2927*Δ1	–	8	118.4
XP106	*tonB7; XCC3205*::pVO155	–	8	82.6
XP107	*tonB8; XCC3967*::pVO155	–	8	91.1
*Xcc*568		–	120	100.0
XP085	*suxC*::pVO	–	120	2.5
XP087	Δ*suxA*	–	120	9.5
*Xcc*568		Sucrose 0,5 µM	120	90.2
*Xcc*568		Sucrose 5 µM	120	51.9
*Xcc*568		Sucrose 50 µM	120	10.5
*Xcc*568		Sucrose 100 µM	120	7.1
*Xcc*568		Fructose 0,5 µM	120	87.3
*Xcc*568		Fructose 5 µM	120	86.4
*Xcc*568		Fructose 50 µM	120	90.6
*Xcc*568		Fructose 100 µM	120	86.2
*Xcc*568		Glucose 100 µM	120	80.9
*Xcc*568		–	240	100.0
XP085	*suxC*::pVO	–	240	6.9
XP087	Δ*suxA*	–	240	23.0

a *Xcc*568: wild type strain.

b Compound added prior to the addition of 0.5 µM [^14^C]sucrose.

c Time after [^14^C]sucrose addition.

d Cells were incubated for 10 minutes at 30°C with 20 µM carbonyl cyanide 3-chlorophenyl-hydrazone (CCCP) prior to the addition of [^14^C]sucrose.

The involvement of the SuxA TBDR in sucrose transport was then checked by comparing the uptake rate of [^14^C]sucrose into the wild-type strain and into a *suxA* non polar mutant (Δ*suxA*). Sucrose transport was much lower in the Δ*suxA* strain, compared to the wild-type strain (Figure 8B and Table 2): after 8 minutes, it reached only 3.5% of the transport rate into the wild-type strain. When *suxA* was supplied *in trans* on a constitutive expression plasmid, sucrose transport was more efficient and rapid, with a transport rate value at 8 minutes corresponding to 484% of that of the wild-type strain. This result confirms that SuxA is required for sucrose entry into *Xcc*.

We then studied sucrose transport in *suxR* and *suxC* insertion mutants (*suxR*::pVO and *suxC*::pVO, respectively), and in a *suxB* deletion mutant (Δ*suxB*). When the inner membrane transporter encoded by *suxC* was absent, the sucrose transport rate reached only 2.1% of the value obtained for the wild-type strain. Thus, this protein is necessary for sucrose entry into *Xcc*. On the contrary, sucrose transport was greatly enhanced in the *suxR* repressor mutant and in the Δ*suxB* amylosucrase mutant (488% and 241% respectively of the value obtained for the wild-type strain after 8 minutes) (Figure 8C and Table 2). These results confirm the repressor function of SuxR (see below) and suggest that SuxB also has a repressor activity on *suxA* expression. When *suxB* was supplied *in trans* in the Δ*suxB* mutant, the sucrose transport was reduced to about the same level as the wild-type strain (76.4% of the transport rate level of the wild-type strain).

**Figure 9:**
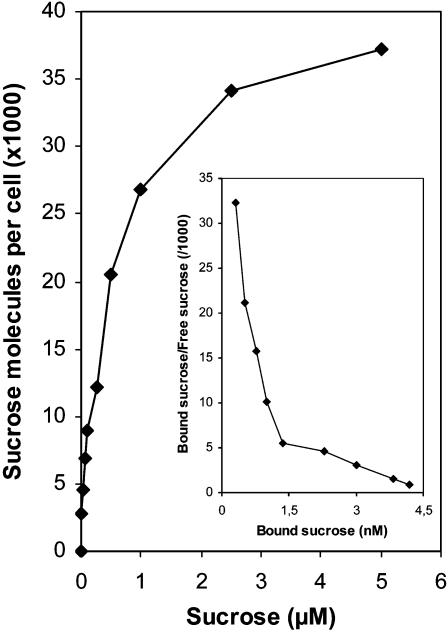
Concentration-dependent [^14^C]sucrose transport into wild-type *Xanthomonas campestris* pv. *campestris*. Cells were grown in minimal medium without sucrose and transport was measured for 15 sec at the indicated [^14^C]sucrose concentrations. The insert shows the Scatchard transformation of the binding data.

Concentration-dependent sucrose transport experiments showed a biphasic kinetics (Figure 9), with a fast rate between 0.01 and 0.1 µM sucrose (*K_d_* = 0.033 µM), and a slow rate between 0.25 and 5 µM sucrose (*K_d_* = 0.59 µM). This biphasic pattern is very similar to those observed in energy coupled transports of vitamin B12 into *E. coli* through the BtuB TBDR [55], [56] and more recently of maltose transport into *C. crescentus* through the MalA TBDR [22]. In both systems, it was concluded that the first phase reflects binding of the transported molecule to the OM TBDR and that the second slower phase reflects binding to a cytoplasmic membrane transporter. Thus, we presume that the low *K_d_* value (0.033 µM) mainly reflects binding to SuxA and that the higher *K_d_* value (0.59 µM) binding to SuxC.

Altogether, these data indicate that both SuxA and SuxC are required for sucrose transport. It is worth noting that if the Δ*suxA* mutant and the *suxC* insertion mutant have a different phenotype regarding their growth ability in presence of sucrose (see figure 7A), both mutants show a very similar alteration in sucrose transport rate after 8 minutes. However, after 240 minutes, sucrose transport rate in the *suxA*-deleted mutant reached almost 23% of the value obtained for the wild-type strain, whereas in the *suxC* insertion mutant, this rate was less than 7% of that of the wild-type strain. This result suggested the existence of two sucrose uptake pathways, an active and a passive pathway, both requiring transport through SuxC. The active transport pathway depends on SuxA for translocation through the outer membrane, whereas the passive pathway does not. It is assumed that the sucrose uptake rate observed into the *suxA*-deleted mutant is determined by slow and passive diffusion of sucrose across the outer membrane, which is sufficient to support growth in the presence of 20 mM sucrose in the medium.

### Sucrose uptake is energy-dependent

Active iron transport through TBDRs depends on the proton motive force (PMF) [56], [57]. Thus, we performed sucrose transport experiments in the presence of a PMF inhibitor, carbonyl cyanide 3-chlorophenyl-hydrazone (CCCP). Addition of 20 µM CCCP inhibited [^14^C]sucrose transport rate to 1.13% of the transport rate in the absence of CCCP (Table 2), demonstrating that sucrose transport through SuxA and/or SuxC is dependent on the proton motive force and does not occur by facilitated diffusion. These experiments did not permit us to conclude whether SuxA-mediated transport depended on the PMF, since SuxC belongs to the Na^+^-melibiose cotransporter and H^+^ cotransporters family, which require the transmembrane potential for transport across the cytoplasmic membrane. We then tested whether TonB was required for sucrose uptake. Eight genes coding for proteins displaying significant similarities to TonB are predicted in the genome of *Xcc* (Table 2). These 8 genes were mutated by insertion of the pVO155 plasmid. As the insertion in *XCC2927* was found to be unstable, we constructed a deletion mutant in this gene (Materials and Methods). Sucrose transport was not significantly impaired in the 8 putative *tonB* mutants (Table 2). This result suggests a complete or partial functional redundancy between at least two *Xcc* TonB proteins. Such a redundancy has already been observed in various other bacteria, e.g. *Serratia marcescens* [58], *Vibrio cholerae* [59] and *Pseudomonas aeruginosa* [60], [61].

### The expression of *sux* genes is induced by sucrose and repressed by SuxR

Using qRT-PCR analyses, we observed that the expression of *suxR*, *suxC* and *suxAB* is specifically induced by the presence of sucrose in the medium (Table 3).

**Table 3:** Relative expression ratios of *suxR, suxC, suxA* and *suxB* in the wild-type strain, in minimal medium containing 20 mM sucrose *vs* minimal medium without sucrose.

Gene name	Ratio (+/− SD) a
*suxR*	5.87 (+/−1.98)
*suxC*	11.14 (+/−0.4)
*suxA*	20.92 (+/−8.5)
*suxB*	44.97 (+/−2.64)

a From real-time quantitative reverse-transcriptase polymerase chain reaction perfomed in at least three independent experiments. Cells were cultivated in minimal medium with or without sucrose. Calculation of relative expression includes normalization against the 16S rRNA endogenous control gene. SD: standard deviation.

As *SuxR* has a high degree of similarity with members of the LacI/GalR family of transcriptional repressors [62], [63], we tested whether this protein regulates the expression of *sux* genes by comparing their expression by qRT-PCR in the *suxR* insertion mutant and in the wild-type strain, cultivated in minimal medium with or without sucrose. *suxAB, suxC* and *suxR* were over-expressed in a *suxR* mutant as compared to the wild-type strain in the absence of sucrose (Table 4), suggesting a negative and effector-dependent control of *sux* genes by SuxR.

**Table 4:** Relative expression ratios of *suxR, suxC, suxA* and *suxB* in the *suxR* insertion mutant (*suxR*::pVO) *vs* wild-type strain.

Gene name	Ratio (+/−SD) a	
	Without sucrose	With sucrose
*suxR*	10.4 (+/−2.3)	1.01 (+/−0.64)
*suxC*	23.6 (+/−4.8)	2.56 (+/−0.8)
*suxA*	154 (+/−83.2)	2.08 (+/−1,8)
*suxB*	176.45 (+/−77.7)	1.54 (+/−0.8)

a From real-time quantitative reverse-transcriptase polymerase chain reaction perfomed in at least three independent experiments. Cells were cultivated in minimal medium with or without sucrose. Calculation of relative expression includes normalization against the 16S rRNA endogenous control gene. SD: standard deviation.

We also studied the expression of the *suxAB* operon in the presence of different sucrose concentrations. For this purpose, the promoter region of this operon was cloned upstream of the promoterless *LacZ* gene in a reporter plasmid (see Materials and Methods). This plasmid, named pPr-*suxA*, was introduced into the *Xcc* wild-type strain, the *suxR* and *suxC* insertion mutants (*suxR*::pVO and *suxC*::pVO, respectively), and the *suxA* and *suxB* deletion mutants (Δ*suxA* and Δ*suxB*, respectively).

**Figure 10:**
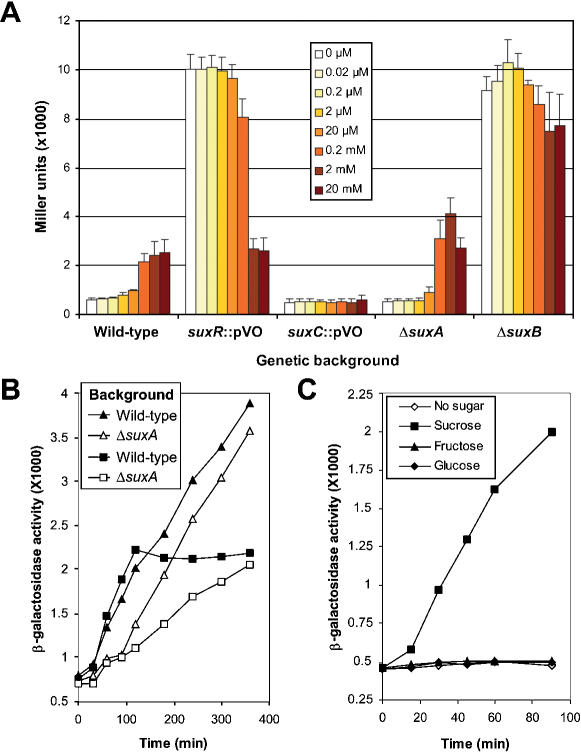
Expression of the *Xanthomonas campestris* pv. *campestris suxA* gene in the presence of sucrose, fructose or glucose. The pPr-*suxA* plasmid carrying the promoterless *LacZ* reporter gene under the *suxA* promoter region was used to monitor *suxA* expression in different genetic backgrounds. Expression was measured in minimal medium and cells were harvested at the indicated times. (A) Expression after 6 hours induction in the presence of different sucrose concentrations, in the wild-type background, the *suxR* and *suxC* insertion mutant backgrounds *(suxR*::pVO and *suxC*::pVO, respectively), and the *suxA* and *suxB* deletion mutant backgrounds *(*Δ*suxA* and Δ*suxB*, respectively). (B) Kinetics of *LacZ* expression in the wild-type background or in the *suxA* deletion mutant background (Δ*suxA*), in presence of 20 mM (triangles) or 100 µM (squares) sucrose. (C) Kinetics of *LacZ* expression in the wild-type background in the presence of 100 µM sucrose, fructose or glucose.

These strains were used to perform β-galactosidase assays after 6 hours growth in minimal medium containing a range of sucrose concentrations (Figure 10A). Induction of the expression of the reporter gene was detected for sucrose concentrations ranging from 20 µM to 20 mM. Maximal induction (3.5 to 4-fold induction) was observed for concentrations higher than 200 µM in the wild-type background (Figure 10A). As expected, the expression of *suxA* was highly induced in the *suxR* mutant, confirming the repressor activity of this gene. However, we observed a significant diminution of the induction level at 2 and 20 mM sucrose in this mutant, suggesting the existence of a second level of control. A high and constitutive induction of *suxA* expression was also observed in the *suxB* deletion mutant, suggesting that the amylosucrase gene plays a role in the repression of the *suxAB* operon. At 2 mM sucrose concentration, a reduction in *suxA* expression level was also observed in this mutant, but it was smaller than that detected in the *suxR* mutant. The deregulation of *suxA* expression in the *suxB* mutant is in agreement with transport studies which showed a higher sucrose transport rate in this mutant (Figure 8C). No induction of *suxA* expression was observed in the *suxC* mutant, whatever sucrose concentration was used. This lack of induction certainly reflects the absence of sucrose entry into the cytoplasm of this mutant, thus preventing the alleviation of SuxR repression. This result clearly shows that the induction of *suxA* expression by sucrose requires the entry of this molecule into the cell. The pattern and level of expression of *suxA* observed in the *suxA* mutant were very similar to those observed in the wild-type strain, except that this expression was significantly higher in the mutant in the presence of 2 mM sucrose. This difference was reproducibly observed and further investigations are needed to understand this phenomenon.

Expression assays suggested that sucrose transport through SuxA is not high enough to influence the expression of the *suxAB* operon. Sucrose entry across the outer membrane by slow diffusion seems to be sufficient to promote induction of *sux* genes after 6 hours growth in the presence of sucrose.

### *suxA* is required for its rapid induction as revealed by using low sucrose concentrations

We performed time-course experiments comparing *suxA* expression in the wild-type strain and the *suxA* deletion mutant grown in presence of different sucrose concentrations (0, 20 µM, 50 µM, 100 µM, 200 µM 500 µM, 1 mM, 2 mM, 5 mM and 20 mM). For clarity, only results obtained with 100 µM and 20 mM are shown in Figure 10B. In the presence of 20 mM sucrose (triangles on Figure 10B), *suxA* expression was not significantly different after 6 hours growth in the wild-type and the *suxA* mutant backgrounds. However, a slight difference was observed in the induction level of *suxA*, from 60 to 120 minutes after sucrose addition. The expression level of *suxA* was 1.5 to 1.6 fold higher in the wild type strain than in the *suxA* mutant. Later, this difference tended to decrease and the ratio reached a value of 1.1 after 6 hours. A reproducible and clear difference in curve profiles was noticed in the presence of 100 µM sucrose (squares on Figure 10B). A rapid induction was observed in the wild-type context, from 30 to 120 minutes after sucrose addition, followed by a plateauing, in which expression seems to remain constant. On the other hand, in the *suxA* deletion mutant background, the induction was linear and less rapid. Between 30 and 120 minutes after sucrose addition, the expression level of *suxA* was 2 fold higher in the wild-type strain background than in the *suxA* deletion mutant background, although after 360 minutes, the expression levels were identical in both strains. Similar results were obtained with sucrose concentrations ranging from 20 to 200 µM (data not shown). We propose that these results could reflect the existence of two pathways controlling *suxA* expression, which can be clearly differentiated with low sucrose concentrations. One of these pathways is controlled by SuxA. These results can be related to the transport analyses showing the existence of two types of sucrose transport across the outer membrane, one being SuxA-dependent and the other one, slower, being dependent on passive diffusion. We thus postulate that the induction of *suxA* indirectly reflects these two means of transport. Moreover, this induction is specific to sucrose and is not due to its degradation products glucose and fructose, as no induction was observed following addition of 100 µM of these molecules in the wild-type background (Figure 10C). Therefore, these experiments suggest that SuxA plays a role in sucrose transport which might be crucial at low concentrations of this sugar.

### Identification of other *Xcc* TBDRs in *loci* probably involved in plant carbohydrates utilization

**Figure 11:**
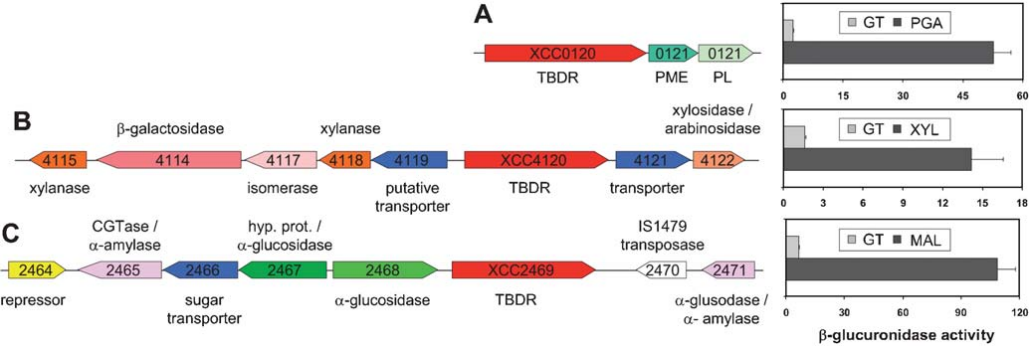
Genetic organization of putative *Xanthomonas campestris* pv. *campestris* CUT loci and specific induction of the TonB-dependent receptor gene by plant carbohydrates. Putative pectin (A), xylan (B) and maltodextrin (C) utilization CUT loci and specific induction of the corresponding TBDR gene in the presence of PGA, xylose and maltose, respectively. β-glucuronidase assays were performed in at least two independent experiments with TBDR insertion mutants cultivated in minimal medium supplemented with glutamate 20 mM (GT) or specific carbohydrates (PGA: polygalacturonic acid 0.125%; XYL: xylose 20 mM; MAL: maltose 20 mM). TBDR: TonB-dependent receptor; PME: pectin methyl esterase; PL: pectate lyase; CGTase: cyclomaltodextrin glucanotransferase.

The genome of *Xcc* was then explored to see whether other TBDRs could belong to loci involved in the utilization of other plant compounds. *Xcc* has an extensive repertoire of plant cell-wall degrading enzymes, with cellulolytic, pectinolytic and hemicellulolytic activities [14]. The analysis of carbohydrate active enzymes identified in the predicted proteomes of 209 Gram negative bacteria and referenced in the CAZy database (http://afmb.cnrs-mrs.fr/CAZY/) showed that, after *Bacteroides* sp. and *S. degradans*, which are well known specialists for polysaccharide degradation, *Xcc* has one of the highest number of genes involved in polysaccharide metabolism per megabase (29.9, total 152) (Table S2). These genes encode 82 predicted glycosyl hydrolases, 45 glycosyl transferases, 5 polysaccharide lyases, 18 carbohydrate esterases and 2 carbohydrate binding proteins. Interestingly, 46 of these proteins are encoded in the vicinity of 24 TBDR/Ps-TBDR genes, thus suggesting the existence of 19 new loci putatively involved in carbohydrate utilization (Table S3). Among those loci, 7 also contain an inner membrane transporter coding gene and a regulatory gene. For 3 loci identified in this analysis, the substrate probably utilized and transported could be easily deduced from the nature of the degradative enzymes: the *XCC0120* TBDR gene is localized upstream of genes coding for a pectin methyl esterase and a pectate lyase and might belong to a locus involved in pectin utilization (Figure 11A); the *XCC4120* TBDR gene is found in a cluster of genes probably involved in xylan metabolism (Figure 11B); and the *XCC2469* TBDR gene might be related to maltose/maltodextrin utilization, since it is associated with genes coding for a cyclomaltodextrin glucanotransferase, two α-glucosidases, a sugar transporter and a maltose transport gene repressor (Figure 11C).

To verify whether these TBDRs are really associated with carbohydrate utilization, we studied their regulation by plant compounds. For this purpose, we performed β-glucuronidase expression assays using all *Xcc* TBDR pVO155 insertion mutants. Cells were grown in rich medium or in minimal medium supplemented or not with polygalacturonic acid (PGA), arabinose, glucose, maltose, sucrose, xylose or xylan. Most TBDR (or Ps-TBDR) genes (44 out of 74) were repressed in rich medium, compared to minimal medium (Table S4). We also observed that in minimal medium, 48 *Xcc* TBDR genes are repressed in the presence of sucrose, xylose, arabinose and/or glucose, suggesting that these genes are submitted to catabolic repression (Table S4). However, the repression patterns were variable, suggesting the existence of several repression pathways. It is worth noting that most Fur-regulated TBDR genes were submitted to catabolic repression.

Three TBDR/Ps-TBDR genes were induced by PGA (*XCC0120, XCC1749* and *XCC1750-1751*), one by maltose (*XCC2469*), seven by arabinose (*XCC0050, XCC1749, XCC1750, XCC1892, XCC2828, XCC4120* and *XCC4222*), three by xylan (*XCC2828, XCC4120* and *XCC4237*) and finally two by xylose (*XCC2828* and *XCC4120*) (Table S4). Interestingly, the 2 TBDRs induced by xylose were also induced by xylan and arabinose, and *XCC1749* and *XCC1750* were both induced by PGA and arabinose. *suxA* is the only TBDR gene for which expression is specifically induced by sucrose, and *XCC2469*, the orthologue of *C. crescentus malA* TBDR gene [22], is the only one induced by maltose. It is worth noting that the expression of the *XCC0120, XCC4120* and *XCC2469* TBDR genes was specifically induced by the postulated substrate of the associated CAZy referenced enzymes (see Figure 11). Altogether, these data suggest that several TBDRs are part of loci which seem to be involved in carbohydrate utilization. We thus propose the existence of 6 putative loci named carbohydrate utilization containing TBDRs (CUT) loci, which were defined by the presence of genes coding for carbohydrate degradative enzymes, inner membrane transporters and sugar related regulators beside TBDR genes. We also identified 15 putative partial CUT loci in the *Xcc* genome (see Table S3).

### Conservation and distribution of TBDRs and CUT *loci* in Xanthomonads

When compared with proteins in the databases using BlastP and the “distance tree of results option” displayed on the BlastP result page, the best homologous genes of *Xcc* TBDRs/Ps-TBDRs were putative TBDRs of other *Xanthomonas* species and in some cases of *Xyllela* strains. These conserved genes might be considered as orthologs. We compared the distribution of *Xcc* TBDRs among Xanthomonads strains for which genome sequences are available, i.e. *Xcc* strain 8004, *Xcv* (strain 85–10), *Xac* (strain 306), *Xoo* (strains KACC10331 and MAFF311018) and *Xf* (strains 9a5c and PD). A search similar to that used for *Xcc* allowed us to identify 72 TBDR/Ps-TBDR in the proteome of *Xcc* strain 8004, 74 in that of *Xac*, 61 in *Xcv*, 41 and 42 in *Xoo* strains KACC10331 and MAFF311018 respectively, and finally 10 in each *Xf* strain. To further study the relationship between these TBDRs and *Xcc* TBDRs, we performed a comparative study using alignments generated by ClustalW [64]. To carry out this comparative analysis, we only used TBDRs which seemed to be complete and Ps-TBDRs over 600 amino acids, in order to avoid bias in alignments. Examination of the phylogenetic tree, coupled with Blast results analysis, clearly confirmed that most *Xcc* TBDRs are well conserved in other *Xanthomonas* strains (Figure S1 and Table S3). All TBDR or Ps-TBDR genes detected in *Xcc* strain ATCC33913 are present in strain 8004; 55 seem to have orthologs in *Xac*; 49 are conserved in *Xcv* and 34 only are conserved in *Xoo* strains which possess significantly less TBDRs. In most instances, the grouping and distribution pattern of orthologous TBDRs matched the phylogenetic classification of *Xanthomonas* species [65]–[67]. This observation suggests that this protein family is ancient in the *Xanthomonas* genus. It is worth noting that branch lengths were variable, suggesting differential evolution rates. Moreover, we noticed that some truncated *Xcc* Ps-TBDRs could be functional in other strains: the Ps-TBDR XCC1750-1751 doublet corresponds to unique and complete TBDR proteins in *Xcv, Xac* and *Xcc* strain 8004. Similarly, the 2 Ps-TBDRs XCC3215-3216 and XCC3270-3271, which are also truncated in *Xcc* strain 8004, correspond to unique proteins in both *Xac* and *Xcv*. Interestingly, *Xac, Xcv* or *Xoo* TBDR orthologs of *Xcc* Ps-TBDR having non-canonical C-terminal regions (β-sheet ended by an aromatic amino acid followed by a short extension of 1 to 4 amino acids), also displayed this feature (data not shown). In most cases, these non canonical C-terminal domains were identical in all strains. This conservation might reflect a specific functional feature of *Xanthomonas* TBDRs.

The analysis of the phylogenetic tree also showed that Xanthomonads TBDRs can be divided into three main groups, with most TBDRs belonging to group 1. This group can be divided into 8 subgroups (1A to 1H). We noticed that (i) *Xcc* Oar-TBDRs are clustered in subgroup 1D, (ii) most plant carbohydrate induced TBDRs (8 out of 11) are grouped in subgroup 1C, and (iii) most *Xcc* Fur-regulated TBDRs (6 out of 8) are grouped in subgroup 1F. Among these Fur-regulated TBDR genes, *XCC3518*, *XCC3595* and *XCC4162*, which belong to subgroup 1F, seem specific to *Xcc* strains.

The comparison of genes located adjacent to orthologous TBDR genes in the different *Xanthomonas* strains showed that in most cases, these regions are syntenic (Table S3). Thus, all putative CUT loci identified in *Xcc* genome are well conserved in *Xac* and *Xcv* but only 2 out of 6 were found in *Xoo*. Similarly, almost all putative partial CUT loci are present in both *Xcv* and *Xac*, but only 9 out of 15 are conserved in *Xoo* strains. The putative polygalacturonate utilization locus (*XCC0120-XCC0122*) seems unique to *Xcc*. The genes bordering this locus are conserved and contiguous in *Xcv* and *Xac*, thus suggesting insertion or deletion events (Table S3).

Finally, among the 10 TBDRs identified in *Xf* strains, 6 are conserved in *Xcc, Xac, Xcv* and *Xoo*, 1 is conserved in *Xcc, Xac* and *Xcv*, whereas only 1 is present in *Xcc*. The 2 remaining *Xf* TBDRs (XF0339/PD1711; XF0599/PD1552) seem more divergent and thus specific to *Xf* strains. However, XF0599 displays weak similarities with XCC2772, a Fur-regulated TBDR of *Xcc*. None of the other *Xf* TBDRs are related to *Xcc* Fur-regulated TBDRs nor to *Xcc* TBDRs belonging to putative CUT loci identified in this study. Only 2 *Xf* TBDR genes (*XF2713* and *XF1036*) correspond to *Xcc* TBDRs present in partial CUT loci.

### *Xcc* TBDRs are conserved in aquatic bacteria and/or phytopathogenic bacteria

For each *Xcc* TBDR included in the phylogenetic study, we analyzed the BlastP results obtained on the nr databank, to characterize the next best homologous genes after Xanthomonads orthologs. This analysis allowed us to cluster *Xcc* TBDRs on the basis of the bacterial origin of the homologous TBDR. Thus, *Xcc* TBDRs could be divided into 4 main groups (Table S5). The first group corresponds to TBDRs showing their next best homologies (after Xanthomonads orthologs) with other *Xanthomonas* TBDRs rather than with TBDRs from other genera. This observation mainly concerned TBDRs found in subgroups 1B, 1C and 1D of the phylogenetic tree. Their positions in the tree suggested that they could be generated by successive duplication events that occurred before *Xanthomonas* speciation (Figure S1). The second group corresponds to TBDRs which display high similarities with TBDRs of β-Proteobacteria and/or *Pseudomonas* species. Interestingly most of these TBDRs are clustered in subgroup 3 of the phylogenetic tree. Moreover, some of these TBDRs showed high similarities with TBDRs of phytopathogenic bacteria such as *Acidovorax avenae* subsp. *citrulli, Pseudomonas syringae* pathovars or *Ralstonia solanacearum*. The third group contains two *Xcc* TBDRs proposed to be involved in iron uptake and showing significant similarities with putative TBDRs from *Cyanobacteria*. The fourth group, which is the largest one (with 35 TBDRs out of 70), corresponds to TBDRs which displayed very significant homologies with putative TBDRs of a wide range of aquatic bacteria belonging to the α or γ classes of Proteobacteria. It is worth noting that in most cases, *Xcc* TBDRs were not affiliated to TBDRs of a specific bacterial class, but there was rather a variety of origins. Thus, for 16 *Xcc* TBDRs the best similarities (after Xanthomonads similarities) were obtained with putative TBDRs of *C. crescentus* CB15 and *Caulobacter* sp. K31 strains, which are aquatic oligotrophs belonging to the Caulobacterales order of the α-Proteobacteria. Homologies were also observed with TBDRs of other α-Proteobacteria living in aquatic habitats, such as *Oceanicaulis alexandrii, Maricaulis maris* or *Parvilarcula bermudensis* HTCC2503, or found in multiple environments like *Sphingomonas* sp. SKA58, *Sphingopyxis alaskensis* or *Novosphingobium aromacitovorans*. Significant similarities were also obtained with putative TBDRs of aquatic γ-Proteobacteria classified in the *Alteromonadales*, including *Alteromonas macleodii, Saccharophagus degradans* 2–40, *Pseudoalteromonas* and *Shewanella* species. One common trait of most of these bacteria is that they show TBDRs overrepresentation with values of TBDR number per megabase ranging from 7.4 to 15.7 (Table S2). This raised the question of whether homologies between these TBDRs were fortuitous and a consequence of their large number or whether they reflect common biological functions.

### Conservation of TBDR regions and CUT loci in Gram-negative bacteria

Some regions surrounding TBDR genes were found to be conserved in closely related bacteria such as *Vibrio parahaemolyticus* or *P. aeruginosa*. Thus, the XCC3050 Fur-regulated TBDR belongs to a cluster of 6 genes showing similarities with the *pvuA*-*pvsABCDE* gene cluster of *V. parahaemolyticus*, involved in the uptake and biosynthesis of the siderophore vibrioferrin [68] (Table S3). Similarly, a group of proteins encompassing XCC3067 TBDR and putatively involved in cobalamin uptake and biosynthesis in *Xcc* was conserved in *Pseudomonas* species (Table S3). Preliminary experiments showed that the expression of this TBDR gene is repressed by the presence of vitamin B12 in *Xcc*, thus suggesting that this cluster is functional (data not shown).

Several CUT loci identified in *Xcc* were not conserved in taxonomically related bacteria but rather in the group of aquatic bacteria showing TBDR conservation and in particular in *C. crescentus*. The *sux* locus, 3 of the putative CUT loci and 4 of the putative partial CUT loci identified in this study were entirely or partially conserved in this group of bacteria (Table S3). Thus, the putative maltose CUT locus was partially conserved with the *mal* locus recently identified in *C. crescentus*, which contains the MalA TBDR proposed to be involved in the uptake of maltodextrins [22] (Figure S2B). Interestingly, MalA shows significant similarities with the *XCC2469* TBDR whose expression is induced by maltose and maps in the maltose CUT locus (Figure 11C).

Similarly, the putative xylose locus, containing the *XCC2828* TBDR gene, is also very well conserved in *C. crescentus* (Figure S2C). The corresponding *CC0999* TBDR gene was shown to be induced by xylose in this bacterium [69]. Moreover, *CC2832*, which was also shown to be xylose-induced in *C. crescentus*, displays significant homologies with the xylose-induced TBDR gene *XCC4120* belonging to a xylose CUT locus. Furthermore, the 20-bp palindromic motif conserved upstream of genes of the *C. crescentus* xylose regulon, was also found upstream of genes putatively involved in xylose metabolism in *Xcc*, as well as in the promoter region of *XCC2828* and *XCC4120* TBDR genes [69].

The *sux* CUT locus seems less well conserved and showed some degree of variation (Figure S2D). The amylosucrase gene (*XCC3359*) is only well conserved in the corresponding *C. crescentus* locus. In the loci conserved in other bacteria, the degradation of sucrose seems to involve more classical pathways (for review see [70]). Moreover, the MFS transporter of the *Xcc* locus is different from that of the *C. crescentus* sucrose locus encoded by *CC1133*, although it is well conserved in both *Sphingomonas* SK58 and *Erythrobacter litoralis*. These differences show the existence of some degree of plasticity in the evolution of the putative CUT loci. Further investigations are needed to see whether all these partially conserved loci are involved in the utilization of the same molecule. However, this wide conservation of CUT loci is in favor of their existence. These results also confirm that the similarities observed between TBDRs are probably not fortuitous.

## Discussion

### Overview

The outer membrane (OM) of Gram negative bacteria serves as a selective permeation barrier, excluding hydrophilic solutes, including most nutrients. However, OMs contain embedded integral proteins, named outer membrane proteins (OMPs), which allow sensing and entry of nutrients into the cell. A major class of OMP with a certain substrate specificity, called porins, allows the translocation of hydrophilic solutes through the OM. Another class of OMPs, the TonB-dependent receptors (TBDRs), is mainly known to be involved in iron or vitamin B12 uptake [71]. Recently, the MalA TBDR of *C. crescentus* was shown to be involved in the ExbBD-dependent uptake of maltodextrins [22]. Here, we show that several Gram negative bacteria, belonging to different lineages and having diverse habitats, display an overrepresentation of TBDRs, which might be related to the uptake of plant-derived carbohydrates.

### *Xcc* TBDRs belonging to the Fur regulon

Our global study of *Xcc* TBDRs showed that only a small fraction of them seems to be involved in iron uptake. Among the 72 TBDR/Ps-TBDRs identified in the *Xcc* genome, only 9 are up-regulated under iron-limiting conditions. We established that these 9 genes are repressed by the Fur repressor and that they are the only TBDR genes having a Fur-box in their promoter region. Recently, a proteomic approach carried out in *P. aeruginausa* PAO1, which contains 34 putative TBDR genes, identified a very similar number of TBDR genes regulated by iron-stavation. In fact, in this bacterium, 7 TBDRs are produced under iron-starvation conditions and 4 others are specifically induced by the presence of heterologous siderophores, under iron-restricted conditions [72]. Therefore, although it is possible that some *Xcc* TBDR genes need specific heterologous siderophores for their expression, as observed in *P. aeruginosa*, the number of TBDR genes involved in iron uptake seems very comparable in both bacteria. This suggests that the other putative TBDRs/Ps-TBDRs might be required for different biological functions.

### The identification of CUT loci/systems in *Xcc* suggests a relationships between TBDRs and carbohydrate utilization

Our data reveal that several *Xcc* TBDRs are related to plant carbohydrate utilization. A large proportion of *Xcc* TBDR genes are coupled with carbohydrate active enzymes. For 6 of them, an inner membrane transporter gene and a regulatory gene are also present in the same region, thus defining the existence of putative CUT loci involved in the uptake and utilization of plant carbohydrates. Moreover, 15 *Xcc* TBDR genes belong to partial CUT loci, thus suggesting the existence of CUT systems composed of different parts scattered in the genome. The existence of such multipartite CUT systems was supported by the observation that 11 TBDR/Ps-TBDR genes are specifically induced by plant carbohydrates such as sucrose or plant cell wall derived compounds including arabinose, xylan/xylose, pectin/polygalacturonate or maltose. Moreover, in 5 cases, there is a correlation between the inducing carbohydrate and the degradative enzyme(s) present in the CUT locus. This allows us to propose the existence of a sucrose CUT locus as well as loci involved in the utilization of complex carbohydrates such as pectin, xylan or starch.

### The *sux* CUT locus is functional

The existence of functional CUT loci was confirmed by the detailed study of the sucrose CUT locus, which showed its involvement in the entry and utilization of sucrose in *Xcc*. This locus comprises four genes, *suxA, suxB, suxC* and *suxR*, coding for a TBDR, an amylosucrase, a sugar inner membrane transporter and a regulatory protein, respectively. [^14^C]sucrose uptake experiments showed that SuxA and SuxC are both required for sucrose entry into the cell. Concentration-dependent sucrose transport experiments showed a biphasic kinetic, with a similar pattern observed for vitamin B12 uptake into *E. coli* through the BtuB TBDR [55], and more recently for maltose transport into *C. crescentus* through the MalA TBDR [22]. In both of these systems, it has been concluded that the first phase reflects the binding of the transported molecule to the OM TBDR, and that the second slower phase reflects the binding to a cytoplasmic membrane transporter. Thus, we presume in our experiments that the low *K_d_* value (0.033 µM) mainly reflects binding to SuxA and that the higher *K_d_* value (0.59 µM) binding to SuxC. Sucrose transport was significantly lower in the *suxC* mutant than in the *suxA* non polar mutant. This difference might explain the differential phenotype of these two mutants observed for growth on sucrose: the *suxA* non polar mutant was not impaired in growth on media containing sucrose, whereas the *suxC* mutant was unable to grow under these conditions. These observations suggest the existence of an alternative pathway that supports facilitated diffusion of sucrose across the OM, whereas it seems that there is a unique route for crossing the inner membrane, depending on SuxC. The existence of this alternative pathway was also revealed by expression analyses of the *sux* locus. The regulation of *sux* genes seems to follow a classical inducer/repressor control, mediated by the SuxR repressor, sucrose being the inducer. The expression of *sux* genes is induced by sucrose but not by fructose or glucose. This induction was detected with sucrose concentrations ranging from 20 µM to 20 mM. These experiments showed that SuxA transport influenced sucrose induction of *suxA* at low sucrose concentrations (20 to 200 µM), whereas this effect is masked at higher concentrations, probably by interference by passive diffusion.

Our data also indicated that sucrose transport through the *sux* system is active and depends on the proton motive force. However, we could not conclude whether it depends on the TonB-ExbBD energy coupling system. In addition to TonB, the *Xcc* genome harbours 7 TonB-like proteins, which might substitute one for another. Another *Xcc* feature is the presence of a second *exbD* gene, named *exbD2*, mapping downstream of the *tonB-exbBD1* locus. Interestingly, *exbD2* is not required for iron uptake but is essential for HR induction, whereas *tonB*, *exbB* and *exbD1* genes are necessary for both processes [73], [74]. This differential behavior might be related to the existence of at least two classes of TBDRs in *Xcc*, one involved in iron uptake and the other one in transport of plant compounds. Further work is needed to address this hypothesis.

### The *sux* locus represents a new sucrose utilization system

The *Xcc sux* locus is clearly different from other sucrose utilization loci already found in bacteria (for review see [70]). In particular, it differs from the *scr* sucrose-utilization system from enteric bacteria, which is also present in the plant pathogenic bacterium *Erwinia amylovora*, where it plays a major role in plant colonization [75]. The differences concern regulation, sucrose utilization and transport. The *scr* genes are regulated by a LacI/GalR family repressor, but fructose is the inducer [76]. In this system, the degradation of sucrose uses a sucrose-6-phosphate hydrolase/fructokinase degradation pathway [70]. On the contrary, the *Xcc sux* locus contains an amylosucrase orthologous to SUH, a unique sucrose hydrolase previously characterized in *Xag* [54]. This enzyme is responsible for intracellular sucrose hydrolysis. It is active on sucrose but not on sucrose-6-phosphate. In the s*cr* system, sucrose metabolism and uptake are coupled by the phosphoeneolpyruvate-dependent carbohydrate:phosphotransferase system (PTS), which controls crossing of the inner membrane and sucrose phosphorylation. The transport across the outer membrane is mediated *via* a porin, named ScrY [77], [78]. Thus, the transport of sucrose is very different in *sux* and *scr* systems. Interestingly, the *K_d_* value of sucrose binding to SuxA is 1500- to 3000-fold lower than that of the *E. coli* ScrY sucrose porin, which varies from 13 mM [79] to 50 mM [80]. Similarly, *C. crescentus* MalA TBDR transports maltodextrins with *K_d_* values 1000-fold lower than those of the LamB porin, which facilitates the passive diffusion of maltodextrin [22]. It is worth noting that the *K_d_* values of sucrose binding to SuxA and maltodextrins binding to MalA are comparable. Thus, SuxA and MalA represents a new class of outer membrane carbohydrate transporters showing a much higher affinity for their substrate than porins.

The importance of the *sux* locus in *Xcc* is highlighted by the fact that it is required for full virulence on *Arabidopsis thaliana*. The phenotype of *suxB* and *suxC* mutants on plants can be related to their inability to grow on medium containing sucrose (20 mM). However, the non polar *suxA* mutant, which grows as well as the wild type strain on medium containing sucrose, was also affected in pathogenicity. Thus, it appears that the ability to scavenge sucrose with a very high affinity plays a key role during the interaction with host plants.

**Figure 12:**
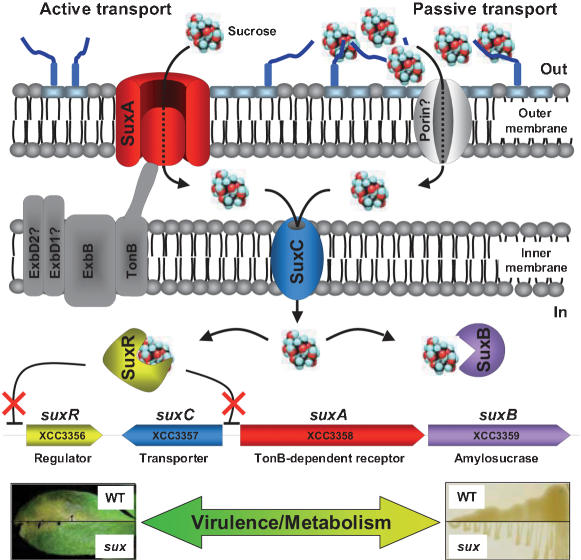
Model of CUT loci functioning based on the *Xanthomonas campestris* pv. *campestris sux* locus. This scheme shows sucrose outer membrane transport *via* the SuxA TBDR or by passive diffusion through a putative porin. After crossing the inner membrane through the SuxC transporter, sucrose is proposed to interact with the SuxR repressor (thus allowing *sux* gene induction) and also to serve as a substrate for the SuxB amylosucrase. The large double headed arrow below the *sux* locus represents the balance between metabolic adaptation and virulence control putatively mediated by CUT loci.

In conclusion, data obtained on this sucrose CUT locus strongly support the existence of the other CUT loci identified in *Xcc*, which might have a similar mode of action (see model presented in Figure 12). This suggests the presence of several systems which seem to partially overlap and which are involved in the scavenging of plant molecules. They might form a complex network required for the exploitation of plant resources but which might also participate in signaling.

### TBDRs and CUT loci are ancient in the *Xanthomonas* genus

The importance of TBDRs and CUT loci is not restricted to *Xcc* since they are well conserved in *Xac* and *Xcv*. A phylogenetic study of Xanthomonads TBDRs suggests that these proteins are an ancient class of proteins in the genus. Moreover, the grouping pattern observed in our phylogenetic study seems to correlate with functional features of *Xcc* TBDRs, thus suggesting the existence of structure/function relationships. However, it seems that there is some degree of variation in the repertoire of TBDR genes and CUT loci among *Xanthomonads*, as the number of genes and loci was lower in *Xoo* strains and also in *Xylella*. This latter species is considered as a minimal pathogen in the Xanthomonads with a restricted habitat and a reduced genome [81]. *Xylella* possesses a significantly lower number of TBDRs and only one partially conserved CUT locus.

### Conservation of *Xcc* TBDRs and CUT loci reveals carbohydrate scavenging abilities in other bacteria

One of the main surprises of this work was the observation that the large majority of *Xcc* TBDRs (35 out of 72) display significant similarities with TBDRs of bacteria which are mostly found in aquatic habitats and which are not closely related to Xanthomonads (see Table S5). Phylogenetic studies place Xanthomonads as a deep branch in the γ-Proteobacteria class, close to that of the β-Proteobacteria [66], [82]. The bacteria possessing similar TBDRs belong either to the α-Proteobacteria class or to the Alteromonadales order of the γ-Proteobacteria. Most bacteria in this latter order have been collected from diverse aquatic environments and belong mainly to 6 different genera: *Alteromonas, Colwellia, Idiomarina, Pseudoalteromonas, Saccharophagus* and *Shewanella*. The α-Proteobacteria mentioned in this study are also found mostly in aquatic habitats and are members of several orders, including Caulobacterales, Sphingomonadales, Rhodospirillales and Rhodobacterales. Similarities were not restricted to TBDRs genes, and genome context analyses showed that at least 8 putative (partial) CUT loci identified in *Xanthomonas* species were conserved among members of these aquatic bacteria. Several of them are found in association with abiotic or biotic surfaces, such as the surface of alga or shellfish. Some are adapted to live in oligotrophic environments, while others are abundant in nutrient-rich habitats or in association with particulate of organic matter. Some others are associated with decaying tissues of plant or algae. Although *Xanthomonas* seems to be very different from these bacteria in terms of habitat, lifestyle and taxonomy, we identified several common traits. All these bacteria belong to the class showing a TBDR overrepresentation (with TBDRs/Mbp ratios>7.4). Most of them are able to degrade a wide variety of plant molecules or other complex carbohydrates such as chitin, alginate, as well as various aromatic compounds. This combination of characters and the similarities with *Xcc* suggest the existence of a shared biology which might be related to the ability to scavenge carbohydrates. If the situation in these bacteria is similar to that observed in *Xcc*, we can speculate that there is a significant proportion of TBDRs involved in the uptake of molecules other than iron-siderophores complexes. Moreover, as previously noticed [33], we observed that bacteria having a restricted habitat, such as obligate parasites or symbionts, have no or only a very small number of TBDRs. Thus, these proteins might be considered as good indicators of bacterial lifestyle.

### Association between TBDRs and carbohydrate utilization in other Proteobacteria

A specific role for TBDRs has already been attributed to bacteria displaying an overrepresentation of this protein family. Sphingomonads are widely distributed in nature and are mostly found in soils and aquatic environments. Some strains are associated with plants [83]. Strains like *Sphingomonas* sp. A1 are able to take up and metabolize macromolecules such as alginate produced by brown seaweed and certain bacteria. This uptake is mediated by large structures, so called superchannels, which form a pit in the outer membrane, and which function as a funnel or concentrator (for review, see [84]). Four TBDRs seem to be part of this superstructure and are thus proposed to participate in alginate transport [85]. Recently, the manipulation of these superchannels revealed the importance of these structures in bioremediation [86].

In *C. crescentus*, beside the prediction of 67 TBDRs, a genome analysis revealed the presence of several genes for the breakdown of plant polysaccharides as well as transport systems, suggesting that plant polymers are a significant source of nutriment for this organism [87]. Interestingly, several *C. crescentus* TBDR genes have been shown or proposed to be associated with the uptake of plant compounds. As described above, Neugebauer and colleagues showed that *C. crescentus* can grow on maltodextrins and that the transport of these molecules is mediated by the MalA TBDR [22]. Proteomic and transcriptomic studies have shown that TBDR genes are expressed at higher levels in minimal medium than in rich medium [69], [88]–[90], as we observed in *Xcc*. Moreover, several TBDR genes are specifically induced by the presence of xylose [69]. Interestingly, 2 TBDRs induced by xylose are conserved between *C. crescentus* and *Xcc*. Strikingly, putative regulatory *cis*-element motifs are conserved in the promoters of these homologous genes in both species. Moreover, one of these 2 *Xcc* TBDR genes (*XCC2828*) belongs to a putative partial CUT locus which is conserved in *C. crescentus* (Figure S2C). This suggests a possible lineage between these loci. Moreover, 3 other *Xcc* CUT loci are entirely or partially conserved in the *C. crescentus* oligotrophic bacterium, including the maltose and sucrose CUT loci. TBDRs in oligotrophs like *C. crescentus* might play a very important role by allowing the foraging of carbohydrates in nutrient poor environments.

### Common themes between *Xanthomonas* and aquatic bacteria

It is clear that the life cycle of *Xanthomonas* spp. presents common features with the different lifestyles of bacteria described above. The leaf surfaces encountered by *Xanthomonas* during their epiphytic development might correspond to an oligotrophic environment. Plant leaves release secondary metabolites which can be used by epiphytic microorganisms [83]. However, carbon resources have been shown to be the most limiting resource on plants [91]. Simple sugars, like glucose, fructose and sucrose, are the most dominant carbon sources on plants that have been examined [92]. Studies with bacterial biosensors *in situ* on plants revealed a high heterogeneity of sucrose availability, with an average accessibility of only about 20 µM on moist bean leaves [93]. Interestingly, this sucrose concentration allowed the induction of *suxA* gene in our expression assays, thus suggesting that the *sux* uptake system might function on plant surfaces. The involvement of *sux* locus in epiphytic life is now being investigated. Furthermore, TBDRs might facilitate the exploitation of plant debris generated during disease development, thus resembling bacteria living on dead tissues. This phenomenon might have an impact on *Xanthomonas* life cycle by increasing *Xanthomonas* population size and thus facilitating new infections.

### TBDRs and virulence on plants

It appears that in *Xanthomonas*, some TBDRs such as SuxA play an additional and specific role by controlling virulence. Therefore, it is tempting to propose that a strategy shared with non-pathogenic bacteria and based on the active uptake of plant-derived nutrients, could have been diverted by *Xanthomonas* for the control of pathogenicity. It is worth noting that several phytopathogenic bacteria such as *P. syringae* or *E. carotovora* subsp. *Atroseptica*, as well as *Pseudomonas* species associated with plants, have an intermediate overrepresentation of TBDRs, suggesting that this feature could be shared by other bacteria interacting with plants. In *R. solanacearum*, the expression of *hrp* genes coding for a type III secretion system (TTSS) which controls disease and HR development, is specifically induced upon contact with plant cells [94]. This signaling is mediated by PrhA, a TBDR belonging to the transducer subclass. This receptor senses contact with plant cells and transduces this signal into the cytoplasm via PrhI and PrhR, which are FecI/FecR homologs [94]–[96]. Apparently, this regulatory system does not require the transport of plant molecules [94]. Although *Xac, Xcc, Xoo* or *Xcv* do not carry any TBDR showing a high homology with PrhA, recently a TBDR controlling both HR and pathogenicity was described in *X. oryzae* pv. *oryzicola*, the causal agent of bacterial leaf streak of rice. This TBDR does not belong to the transducer subclass and only shows a weak similarity with PrhA [97]. This TBDR gene is highly conserved in *Xoo* strain MAFF 311018 (*XOO0785*), *Xcv* (*XCV3654*) and *Xcc* (*XCC0674*). Interestingly, in *Xoo* it is located close to *trh* (*XOO0783*), a regulatory gene controlling the expression of *hrp* genes [53]. The orthologue of this regulatory gene is also conserved in *Xcc* (*XCC0672*), in the vicinity of the *XCC0674* TBDR gene. Thus, it is possible to speculate that both genes might be involved in a common circuit controlling the expression of *hrp* genes in *Xanthomonas* spp. In our study, a mutation in *XCC0674* did not affect the pathogenicity of *Xcc*. However, as there are more TBDRs in *Xcc* than in *Xoo* strains, we can speculate that functional redundancy might have masked the effect of the mutation. Further work is needed to see whether these genes regulate *hrp* genes in *Xcc*. Nevertheless, we established a link between *hrp* genes and TBDRs in *Xcc*. Indeed, we observed that 2 *Xcc* TBDR genes are regulated by the *hrpG* and *hrpX* regulatory genes. Therefore, it seems that there is an overlap between *hrp* genes and at least 2 TBDR genes. What is the function of these two TBDRs? Are they specifically required during the infection of plants, through the action of the *hrp* regulon, to exploit specific released plant molecules?

### What other functions for *Xcc* TBDRs?

We have been able to define a putative role for 21 TBDRs out of 72 identified in *Xcc*. The nature of the molecules putatively transported by the other TBDRs remains to be discovered. It is probable that TBDRs are not restricted to the transport of carbohydrates and that they can take up various other molecules produced by plants. *Azospirillum irakensis*, a plant associated bacterium, is able to metabolize salicin, a phenolic glycoside produced by plants. Interestingly, the *sal* operon, which controls the degradation of salicin, contains a TBDR gene which was proposed to be involved in salicin uptake [98]. Thus, secondary metabolites including phenolic compounds might be assimilated through TBDRs. We are now trying to characterize which new molecules may be transported by *Xcc* TBDRs, to better understand the adaptation of this pathogen to host plants.

### TBDRs, CUT loci and evolution in Gram-negative bacteria

This work has identified the existence of an ensemble of bacteria that have an overrepresentation of TBDR genes, and that share specific loci for the scavenging and utilization of carbohydrates. They belong to very different lineages in Proteobacteria and this raises the question of the origin of these TBDRs and CUT loci. Did they arise by convergent evolution or were they transferred from species to species by lateral gene transfer? Our data suggest that this latter hypothesis is most likely. Recently, a genomic comparative study established that *Xcc* and *Xac* have close to 40% of their genes showing highest similarities to genes from non γ-Proteobacteria, especially from α-Proteobacteria (20%). These genes seem to belong to genomic islands, denominated “unusual best-match islands” (UBIs) [82]. Interestingly, among 35 UBIs thus identified in *Xcc* genome, 14 contain TBDRs and/or CUT loci. However, there was no significant difference between the GC content of each of these loci and the overall content of the genome. It is therefore possible that these loci were acquired very early in the evolution of Xanthomonads and thus played a key role in their adaptation to plants. Most bacteria showing a TBDR overrepresentation possess TBDRs or CUT loci conserved with *Xcc*. However, there is a main exception with members of the Bacteroidetes phylum, as none of the TBDRs characterized in this phylum showed very significant similarities with those identified in *Xcc*. Bacteroidetes can be encountered in two very different niches, the marine environment and the human intestine [99], [100]. Marine Bacteroidetes such as *Gramella forsetii* are associated with particulate of organic matter [99], whereas those found in the intestine are assembled on partially digested food particles [101]. In both cases, these bacteria are able to consume biopolymers. *Bacteroides thetaiotaomicron*, which is a prominent mutualist in the distal intestine of adult humans, has the largest ensemble of TBDR genes and glycobiome yet reported (Table S2). Studies have shown that this bacterium has a carbohydrate foraging behavior [100]–[102]. It is well known to bind starch through a protein complex of the outer membrane, which comprises SusD and the SusC TBDR protein [103]. One hundred and six paralogs of SusC and fifty three paralogs of SusD were predicted in the *B. thetaiotaomicron* genome [104]. In our study, we did not detect in *Xanthomonas* genomes any protein displaying similarities with SusD. Moreover, none of the TBDRs identified in *Xcc* showed strong similarities with SusC. A Blast analysis suggested that the SusC TBDR and the SusD OMP are specifically conserved in Bacteroidetes (data not shown). These results suggest that TBDRs involved in carbohydrate uptake evolved independently in Proteobacteria and Bacteroidetes. The analysis of the function and evolution of TBDRs in these phyla will certainly help us to better understand the adaptation of bacteria to their environment. This knowledge, which concerns the utilization of plant molecules that are widespread in the environment, will have a major impact not only in plant pathology, but also in human health as well as in the cycling of carbon and geobiology in marine environments.

## Materials and Methods

### Bacterial strains, plasmids and growth conditions

The *Xanthomonas campestris* pv. *campestris* (*Xcc*) strains and plasmids used in this study are listed in Table S6. *Xcc* cells were grown at 30°C in MOKA rich medium (Yeast Extract 4 g/l, Casamino acids 8 g/l, K_2_HPO_4_ 2 g/l, MgSO_4_.7H_2_O 0.3 g/l) or in MME minimal medium [12]. *E. coli* cells were grown on LB medium [105]. Antibiotics were used at the following concentrations for *Xcc*: rifampicin, 50 µg/ml; kanamycin: 50 µg/ml; tetracycline: 5 µg/ml. Antibiotics were used at the following concentrations for *E. coli*: ampicillin, 50 µg/ml; kanamycin: 50 µg/ml; tetracycline: 10 µg/ml.

Growth curves were generated using the Bioscreen C instrument (Labsystems, Helsinki, Finland) in three independent experiments. Growth measurements were realized in 200-well microtiter plates on 350 µl volumes of a minimal medium containing 20 mM sucrose, inoculated at an OD_600_ = 0.15 from a washed starter culture. Non-inoculated wells were used as asepsis controls. Optical densities at 600 nm values were measured every 30 min over a period of 2 to 3 days at 28°C. The microplates were shaken for 5 sec before each measurement.

### Construction of *Xanthomonas campestris* pv. *campestris* mutants

Insertion mutants were constructed using the suicide plasmid pVO155 [36]. Oligonucleotide primers used for PCR amplification will be provided upon request. Amplicons were 300 bp in average. Location of insertions are indicated in Table S6. Deletion mutants in *XCC3358, XCC3359, XCC1990, XCC3209* and *XCC2927* were constructed using the *cre-lox* system adapted from Marx and colleagues [37], [38]. Deleted regions are indicated in Table S6.

A *fur* mutant strain was obtained using the manganese mutagenesis method [106] in LB medium containing 5 mM MnCl_2_. After incubation for 48 h at 30°C, surviving colonies were harvested for siderophores over-expression on CAS agar plates [107] adapted for *Xanthomonas* (K_2_HPO_4_ 1 g/l; MgSO_4_ 1 mM; Casamino acids 0.15 g/l; (NH_4_)_2_SO_4_ 1 g/l; sucrose 20 mM; CAS 60.5 mg/l; HDTMA 72.9 mg/l; FeCl_3_ 10 µM). A resistant strain over-expressing siderophores was selected; the *fur* gene was sequenced and contains a point mutation (T212A leading to L71Q).

### Plasmid constructions

The *XCC3358, XCC3359* and *XCC1470* genes (*suxA, suxB* and *fur* respectively, see Figure 6 and Table S6) were amplified by PCR using appropriately designed primers (Oligonucleotide primers used for PCR amplification will be provided upon request). PCR products corresponding to *suxA* and *suxB* genes were cloned into pCZ525, a derivative of pSC154 [108], without *cyaA*' coding sequence. The obtained plasmids were cloned into pLAFR6 [109] to give pL-*XCC3358* and pL-*XCC3359* respectively. The PCR product corresponding to the *fur* gene with its promoter and terminator sequences was cloned into pFAJ1700 [110] to give pL-*XCC1470*.

The *XCC3358, XCC1990* and *XCC3209* promoter regions (see Table S6) were PCR amplified with appropriately designed primers. These promoter regions were cloned as *Hind*III-*Xba*I fragments, into the pCZ750 plasmid, a pFAJ1700 [110] derivative containing the *Kpn*I-*Asc*I *lacZ* gene from the pCZ367 plasmid [108].

### Expression studies

β-galactosidase and β-glucuronidase assays: bacterial cultures in the appropriate medium were harvested at different time points and β-galactosidase and β-glucuronidase assays were performed as previously described [111], [112].

Quantitative RT-PCR (qRT-PCR): a 6 hour bacterial culture in the appropriate medium was harvested at an OD_600_ = 0.4 to 0.6. RNA were extracted using the Rneasy Mini Kit (QIAGEN). One µg of RNA was treated with RNase-free DNase I (GE-Healthcare) for 15 min at 37°C. After DNase inactivation (10 min at 75°C), RNA were reverse-transcribed by Superscript II (Invitrogen) using random hexamers (Biolabs), for 2 min at 25°C followed by 1 h at 42°C. Quantitative-PCR amplification was performed on Light Cycler (Roche): 10 min 95°C, 1 cycle; 10 sec 95°C, 10 sec 65°C, 20 sec 72°C, 40 to 50 cycles). Experiments were carried out in three independent biological experiments. Oligonucleotide primers used for quantitative-PCR amplification will be provided upon request. As a control for real-time PCR, we used the 16S rRNA as described [113]. The 16S rRNA forward primer (5′-TGACGGTACCCAAAGAATAAGCA-3′) and 16S rRNA reverse primer (5′-ACGCTTGCACCCTTCGTATTA-3′) amplicon was 72 bp in length.

### Pathogenicity tests

Pathogenicity tests were conducted on *Arabidopsis thaliana* Sf-2 ecotype as previously described [114]. Each strain was tested on sets of 4 plants with 4 leaves per plant. Disease development was scored at days 5, 7 and 9 post-inoculation using a disease index ranging from 0 (no symptom), to 4 (leaf death).

### [^14^C]sucrose transport experiments

Overnight cultures in minimal medium (MME) without carbon source (uninduced) or with 20 mM sucrose (induced) were centrifuged. Pellets were resuspended in MME and the OD_600_ was adjusted to 1. [^14^C]sucrose (PerkinElmer, specific activity of 21.8 GBq/mmol) was added to a final concentration of 0.5 µM. For competition experiments, sucrose, fructose or glucose was added to [^14^C]sucrose at a final concentration ranging from 0.5 to 100 µM. After different times, from 20 sec to 2 hours, samples of 0.2 ml were collected on cellulose nitrate filters, washed with 10 ml water, dried, and finally, the radioactivity was determined in a liquid scintillation counter.

The concentration-dependent initial sucrose transport was determined using the rapid dilution method as described [22]. Cells were precultured in minimal medium without sugar. After centrifugation and adjustment to an OD_600_ of 1, cells were incubated for 15 sec in presence of 0.01, 0.025, 0.05, 0.1, 0.25, 0.5, 1, 2.5 and 5 µM [^14^C]sucrose and 0.2 ml samples were diluted into 5 ml MME supplemented with 0.1 mM sucrose. Cells were collected by filtration, washed with 10 ml MME supplemented with 10 mM sucrose, dried and the radioactivity was determined.

For inhibition of the proton motive force (PMF) with carbonyl cyanide 3-chlorophenyl-hydrazone (CCCP), cells were incubated for 10 minutes at 30°C with 20 µM CCCP prior to the addition of [^14^C]sucrose.

### *In silico* analysis

Location of the signal sequence responsible for the outer membrane localization was determined using the SignalP 3.0 server [115] (http://www.cbs.dtu.dk/services/SignalP/) with default parameters for Gram-negative bacteria.

Comparison alignments used to identify the TonB-box were realized using ClustalW [64] (http://www.ebi.ac.uk/clustalw/) or Multalin [116] (http://prodes.toulouse.inra.fr/multalin/multalin.html) softwares.

β-sheets in the last 50 amino-acids of *Xcc* TBDRs were located using the secondary structure prediction method [117] on the PSIPRED Protein Structure Prediction Server [118], [119] (http://bioinf.cs.ucl.ac.uk/psipred/).

The MotifSampler program [41], [42] was used to identify a motif corresponding to the *Xcc* Fux-box upstream of the 9 Fur-repressed TBDR and Ps-TBDR genes. MotifScanner program [42] or the PatScan pattern matcher software [47] were used with the identified motif to locate all the Fur-boxes in the *Xcc* genome.

Pip boxes (TTCGCN_15_TTCGC) [120] and hrp_II_ boxes (TTCGN_16_TTCG) [121] were identified in the *Xcc* genome using the PatScan software [47] http://www-unix.mcs.anl.gov/compbio/PatScan/HTML/patscan.html).

For phylogenetic analysis, amino acid sequences were aligned and phylogenetic trees were reconstructed by the neighbor-joining method as implemented in ClustalX [122].

## Supporting Information

Figure S1 Phylogenetic tree of the family of TonB-dependent receptor proteins from Xanthomonas campestris pv. campestris strains ATCC33913 and 8004, Xanthomonas axonopodis pv. citri strain 306, Xanthomonas campestris pv. vesicatoria strain 85–10, Xanthomonas oryzae pv. oryzae strains KACC10331 and MAFF311018 and Xylella fastidiosa strains 9a5c and PD.
(0.04 MB PDF)

Figure S2 Genome context of genes associated with TonB-dependent receptors present in putative (partial) CUT loci of Xanthomonas campestris pv. campestris (Xcc) and conservation in non related bacteria. Homologous genes are marked by matching colors. White color indicates non conserved genes. For conserved genes, percentages of identity and similarity to the corresponding Xcc gene are indicated beneath.
(0.24 MB PDF)

Table S1 Regions and domains of Xanthomonas campestris pv. campestris putative TonB-dependent receptors (TBDRs).
(0.04 MB XLS)

Table S2 Distribution of TonB-dependent receptors in 226 sequenced Gram negative bacterial genomes. The EMBL-EBI, Integr8 web portal (http://www.ebi.ac.uk/integr8/EBI-Integr8-HomePage.do) and the NCBI ENTREZ Genome Project database (http://www.ncbi.nlm.nih.gov/entrez/query.fcgi?db = genomeprj) were used to select sequenced bacterial genomes. TonB-dependent receptors (TBDRs) were detected by screening the Pfam database and the UniprotKB protein knowledge database using the two Pfam domains (PF07715, Plug; PF00593, TonB_dep_Rec). Only proteins displaying the two Pfam domains were considered.
(0.08 MB XLS)

Table S3 Genome context of Xanthomonas campestris pv. campestris (Xcc) TonB-dependent receptor (TBDR) genes and definition of putative CUT loci, conservation in Xanthomonads or in other genera, and TBDR conservation in Pseudomonas aeruginosa PAO1.
(0.18 MB XLS)

Table S4 Differential expression ratios of Xanthomonas campestris pv. campestris TonB-dependent receptor (TBDR) pVO155 insertion mutants.
(0.03 MB XLS)

Table S5 Conservation of Xanthomonas campestris pv. campestris (Xcc) TonB-dependent receptors (TBDRs) beside Xanthomonads orthologies.
(0.04 MB XLS)

Table S6 List of plasmids and Xanthomonas campestris pv. campestris strains used or generated in this study.
(0.03 MB XLS)
