# Supplementary material for: Plant Carbohydrate Scavenging through TonB-Dependent Receptors: A Feature Shared by Phytopathogenic and Aquatic Bacteria
Source: PLoS One. 2007 Feb 21;2(2):e224. doi: 10.1371/journal.pone.0000224 (PMC1790865; doi:10.1371/journal.pone.0000224)
Supplement: Figure S1 — Phylogenetic tree of the family of TonB-dependent receptor proteins from Xanthomonas campestris pv. campestris strains ATCC33913 and 8004, Xanthomonas axonopodis pv. citri strain 306, Xanthomonas campestris pv. vesicatoria strain 85–10, Xanthomonas oryzae pv. oryzae strains KACC10331 and MAFF311018 and Xylella fastidiosa strains 9a5c and PD. (0.04 MB PDF) [file pone.0000224.s001.pdf]

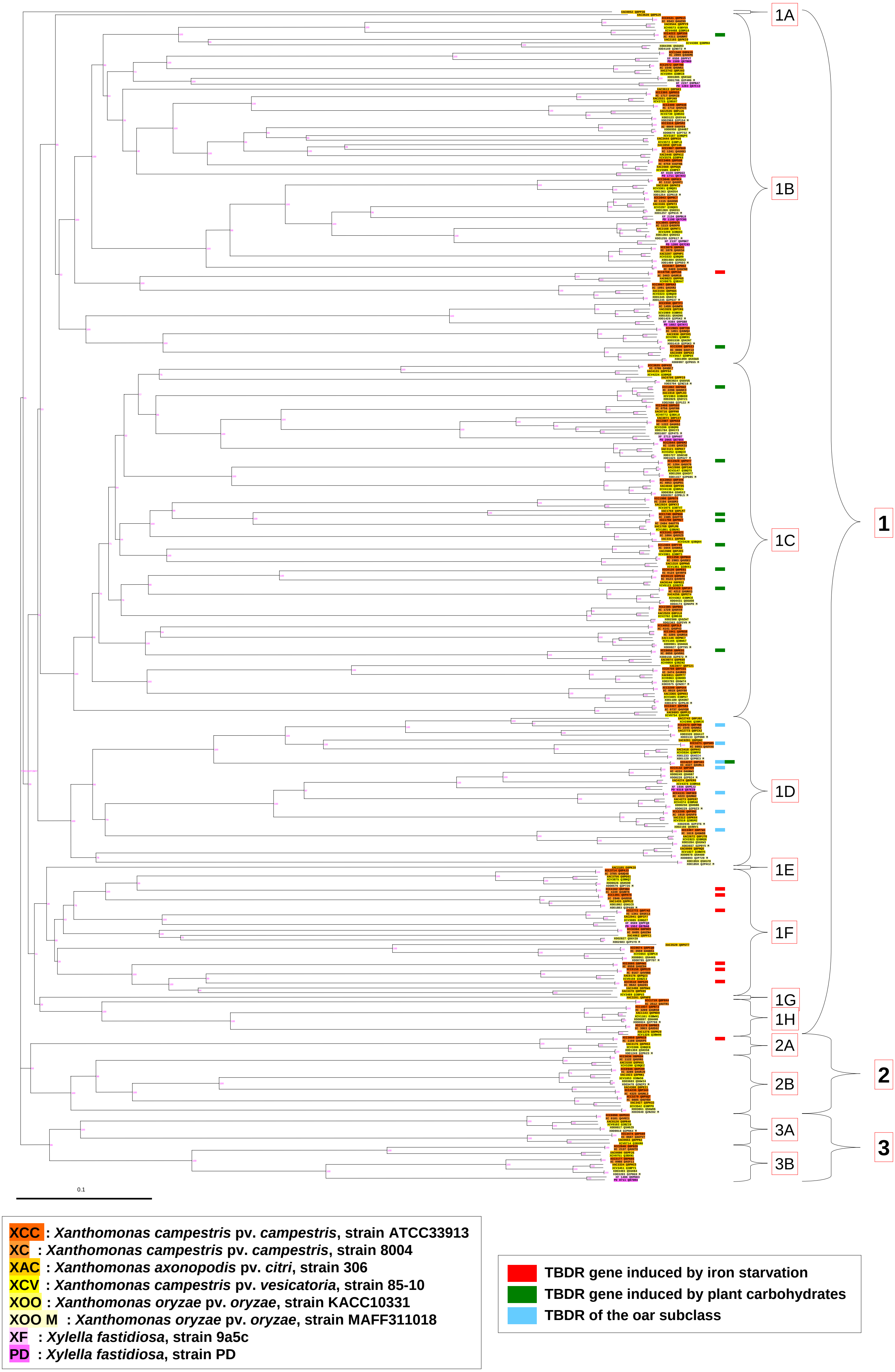
1A
1B
1C
1D
1E
1F
1G
1H
2A
2B
3A
3B
1
2
3
0.1
XCC : Xanthomonas campestris pv. campestris, strain ATCC33913
XC : Xanthomonas campestris pv. campestris, strain 8004
XAC : Xanthomonas axonopodis pv. citri, strain 306
XCV : Xanthomonas campestris pv. vesicatoria, strain 85-10
XOO : Xanthomonas oryzae pv. oryzae, strain KACC10331
XOO M : Xanthomonas oryzae pv. oryzae, strain MAFF311018
XF : Xylella fastidiosa, strain 9a5c
PD : Xylella fastidiosa, strain PD
TBDR gene induced by iron starvation
TBDR gene induced by plant carbohydrates
TBDR of the oar subclass
