# Supplementary material for: Plant Carbohydrate Scavenging through TonB-Dependent Receptors: A Feature Shared by Phytopathogenic and Aquatic Bacteria
Source: PLoS One. 2007 Feb 21;2(2):e224. doi: 10.1371/journal.pone.0000224 (PMC1790865; doi:10.1371/journal.pone.0000224)
Supplement: Figure S2 — Genome context of genes associated with TonB-dependent receptors present in putative (partial) CUT loci of Xanthomonas campestris pv. campestris (Xcc) and conservation in non related bacteria. Homologous genes are marked by matching colors. White color indicates non conserved genes. For conserved genes, percentages of identity and similarity to the corresponding Xcc gene are indicated beneath. (0.24 MB PDF) [file pone.0000224.s002.pdf]

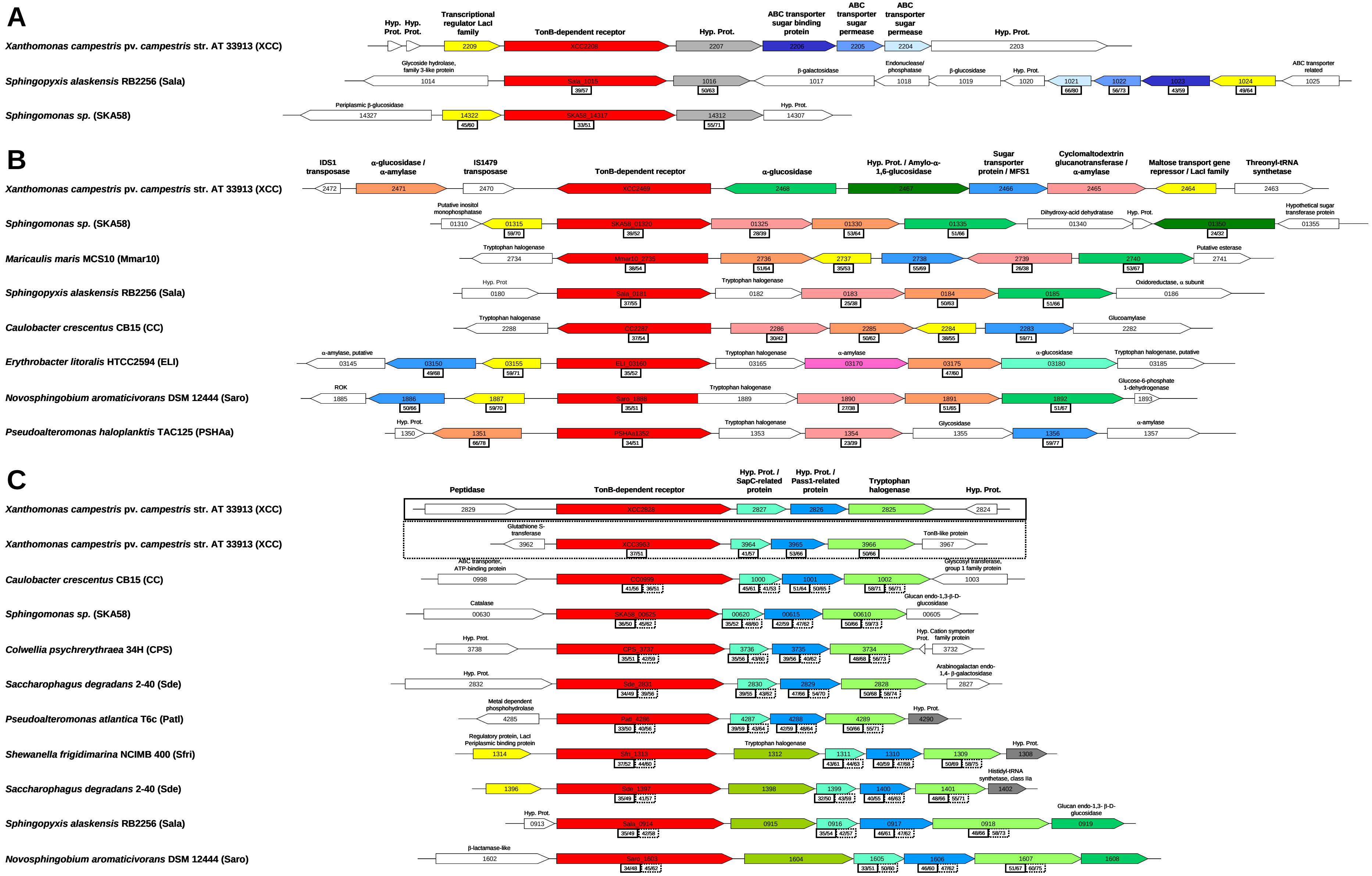
A
Xanthomonas campestris pv. campestris str. AT 33913 (XCC)
Hyp. Prot.
Hyp. Prot.
Transcriptional regulator LacI family
2209
TonB-dependent receptor
XCC2208
Hyp. Prot.
2207
ABC transporter sugar binding protein
2206
ABC transporter sugar permease
2205
ABC transporter sugar permease
2204
Hyp. Prot.
2203
Sphingopyxis alaskensis RB2256 (Sala)
Glycoside hydrolase, family 3-like protein
1014
Sala_1015
39/57
1016
50/63
β-galactosidase
1017
Endonuclease/ phosphatase
1018
β-glucosidase
1019
Hyp. Prot.
1020
1021
66/80
1022
56/73
1023
43/59
1024
49/64
ABC transporter related
1025
Sphingomonas sp. (SKA58)
Periplasmic β-glucosidase
14327
14322
45/60
SKA58_14317
33/51
14312
55/71
Hyp. Prot.
14307
B
Xanthomonas campestris pv. campestris str. AT 33913 (XCC)
IDS1 transposase
2472
α-glucosidase / α-amylase
2471
IS1479 transposase
2470
TonB-dependent receptor
XCC2469
α-glucosidase
2468
Hyp. Prot. / Amylo-α-1,6-glucosidase
2467
Sugar transporter protein / MFS1
2466
Cyclomaltodextrin glucanotransferase / α-amylase
2465
Maltose transport gene repressor / LacI family
2464
Threonyl-tRNA synthetase
2463
Sphingomonas sp. (SKA58)
Putative inositol monophosphatase
01310
01315
59/70
SKA58_01320
39/52
01325
28/39
01330
53/64
01335
51/66
Dihydroxy-acid dehydratase
01340
Hyp. Prot.
01350
24/32
Hypothetical sugar transferase protein
01355
Maricaulis maris MCS10 (Mmar10)
Tryptophan halogenase
2734
Mmar10_2735
38/54
2736
51/64
2737
35/53
2738
55/69
2739
26/38
2740
53/67
Putative esterase
2741
Sphingopyxis alaskensis RB2256 (Sala)
Hyp. Prot
0180
Sala_0181
37/55
Tryptophan halogenase
0182
0183
25/38
0184
50/63
0185
51/66
Oxidoreductase, α subunit
0186
Caulobacter crescentus CB15 (CC)
Tryptophan halogenase
2288
CC2287
37/54
2286
30/42
2285
50/62
2284
38/55
2283
59/71
Glucoamylase
2282
Erythrobacter litoralis HTCC2594 (ELI)
α-amylase, putative
03145
03150
49/68
03155
59/71
ELI_03160
35/52
Tryptophan halogenase
03165
α-amylase
03170
03175
47/60
α-glucosidase
03180
Tryptophan halogenase, putative
03185
Novosphingobium aromaticivorans DSM 12444 (Saro)
ROK
1885
1886
50/66
1887
59/70
Saro_1888
35/51
Tryptophan halogenase
1889
1890
27/38
1891
51/65
1892
51/67
Glucose-6-phosphate 1-dehydrogenase
1893
Pseudoalteromonas haloplanktis TAC125 (PSHAa)
Hyp. Prot.
1350
1351
66/78
PSHAa1352
34/51
Tryptophan halogenase
1353
1354
23/39
Glycosidase
1355
1356
59/77
α-amylase
1357
C
Xanthomonas campestris pv. campestris str. AT 33913 (XCC)
Peptidase
2829
TonB-dependent receptor
XCC2828
Hyp. Prot. / SapC-related protein
2827
Hyp. Prot. / Pass1-related protein
2826
Tryptophan halogenase
2825
Hyp. Prot.
2824
Xanthomonas campestris pv. campestris str. AT 33913 (XCC)
Glutathione S-transferase
3962
XCC3963
37/51
3964
41/57
3965
53/66
3966
50/66
TonB-like protein
3967
Caulobacter crescentus CB15 (CC)
ABC transporter, ATP-binding protein
0998
CC0999
41/56
36/51
1000
45/61
41/53
1001
51/64
50/65
1002
58/71
56/71
Glyscosyl transferase, group 1 family protein
1003
Sphingomonas sp. (SKA58)
Catalase
00630
SKA58_00625
36/50
45/62
00620
35/52
48/60
00615
42/59
47/62
00610
50/66
59/73
Glucan endo-1,3-β-D-glucosidase
00605
Colwellia psychrerythraea 34H (CPS)
Hyp. Prot.
3738
CPS_3737
35/51
42/59
3736
35/56
43/60
3735
39/56
40/62
3734
48/68
56/73
Hyp. Prot.
Cation symporter family protein
3732
Saccharophagus degradans 2-40 (Sde)
Hyp. Prot.
2832
Sde_2831
34/49
39/56
2830
39/55
43/62
2829
47/66
54/70
2828
50/68
58/74
Arabinogalactan endo-1,4- β-galactosidase
2827
Pseudoalteromonas atlantica T6c (Patl)
Metal dependent phosphohydrolase
4285
Patl_4286
33/50
40/56
4287
39/59
43/64
4288
42/59
48/64
4289
50/66
55/71
Hyp. Prot.
4290
Shewanella frigidimarina NCIMB 400 (Sfri)
Regulatory protein, LacI
Periplasmic binding protein
1314
Sfri_1313
37/52
44/60
Tryptophan halogenase
1312
1311
43/61
44/63
1310
40/59
47/68
1309
50/69
58/75
Hyp. Prot.
1308
Saccharophagus degradans 2-40 (Sde)
1396
Sde_1397
35/49
41/57
1398
1399
32/50
43/59
1400
40/55
46/63
1401
48/66
55/71
Histidyl-tRNA synthetase, class IIa
1402
Sphingopyxis alaskensis RB2256 (Sala)
Hyp. Prot.
0913
Sala_0914
35/49
42/58
0915
0916
35/54
42/57
0917
46/61
47/62
0918
48/66
58/73
Glucan endo-1,3- β-D-glucosidase
0919
Novosphingobium aromaticivorans DSM 12444 (Saro)
β-lactamase-like
1602
Saro_1603
34/48
45/62
1604
1605
33/51
50/60
1606
46/60
47/62
1607
51/67
60/75
1608

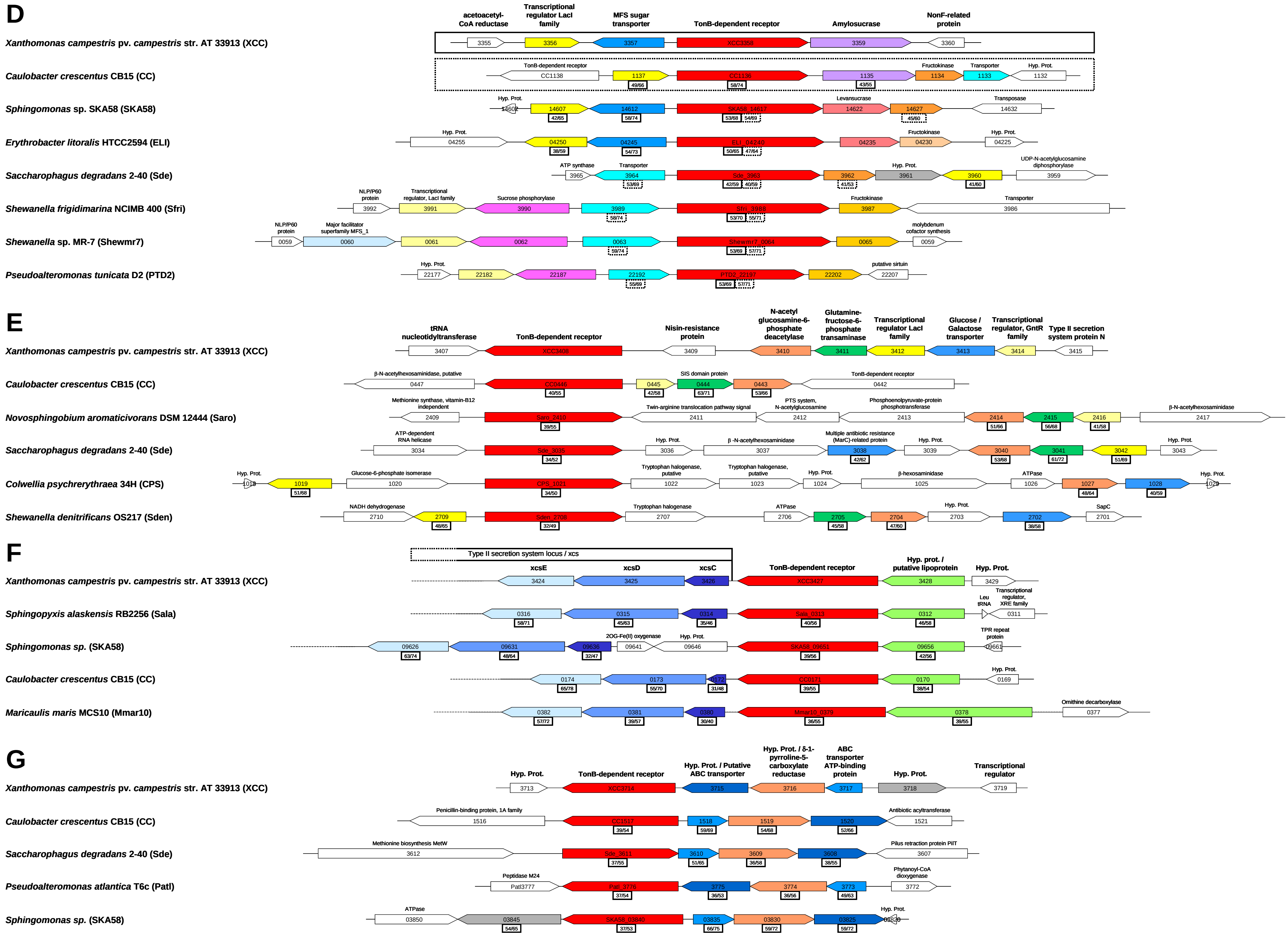
D
acetoacetyl-CoA reductase
Transcriptional regulator LacI family
MFS sugar transporter
TonB-dependent receptor
Amylosucrase
NonF-related protein
Xanthomonas campestris pv. campestris str. AT 33913 (XCC)
3355
3356
3357
XCC3358
3359
3360
Caulobacter crescentus CB15 (CC)
TonB-dependent receptor
CC1138
1137
49/66
CC1136
58/74
1135
43/55
Fructokinase
1134
Transporter
1133
Hyp. Prot.
1132
Sphingomonas sp. SKA58 (SKA58)
Hyp. Prot.
14602
14607
42/65
14612
58/74
SKA58_14617
53/68
54/69
Levansucrase
14622
14627
45/60
Transposase
14632
Erythrobacter litoralis HTCC2594 (ELI)
Hyp. Prot.
04255
04250
38/59
04245
54/73
ELI_04240
50/65
47/64
04235
Fructokinase
04230
Hyp. Prot.
04225
Saccharophagus degradans 2-40 (Sde)
ATP synthase
3965
Transporter
3964
53/69
Sde_3963
42/59
40/59
3962
41/53
Hyp. Prot.
3961
3960
41/60
UDP-N-acetylglucosamine diphosphorylase
3959
Shewanella frigidimarina NCIMB 400 (Sfri)
NLP/P60 protein
3992
Transcriptional regulator, LacI family
3991
Sucrose phosphorylase
3990
3989
58/74
Sfri_3988
53/70
55/71
Fructokinase
3987
Transporter
3986
Shewanella sp. MR-7 (Shewmr7)
NLP/P60 protein
0059
Major facilitator superfamily MFS_1
0060
0061
0062
0063
59/74
Shewmr7_0064
53/69
57/71
0065
molybdenum cofactor synthesis
0059
Pseudoalteromonas tunicata D2 (PTD2)
Hyp. Prot.
22177
22182
22187
22192
55/69
PTD2_22197
53/69
57/71
22202
putative sirtuin
22207
E
tRNA nucleotidyltransferase
TonB-dependent receptor
Nisin-resistance protein
N-acetyl glucosamine-6-phosphate deacetylase
Glutamine-fructose-6-phosphate transaminase
Transcriptional regulator LacI family
Glucose / Galactose transporter
Transcriptional regulator, GntR family
Type II secretion system protein N
Xanthomonas campestris pv. campestris str. AT 33913 (XCC)
3407
XCC3408
3409
3410
3411
3412
3413
3414
3415
Caulobacter crescentus CB15 (CC)
β-N-acetylhexosaminidase, putative
0447
CC0446
40/55
0445
42/58
SIS domain protein
0444
63/71
0443
53/66
TonB-dependent receptor
0442
Novosphingobium aromaticivorans DSM 12444 (Saro)
Methionine synthase, vitamin-B12 independent
2409
Saro_2410
39/55
Twin-arginine translocation pathway signal
2411
PTS system, N-acetylglucosamine
2412
Phosphoenolpyruvate-protein phosphotransferase
2413
2414
51/66
2415
56/68
2416
41/58
β-N-acetylhexosaminidase
2417
Saccharophagus degradans 2-40 (Sde)
ATP-dependent RNA helicase
3034
Sde_3035
34/52
Hyp. Prot.
3036
β -N-acetylhexosaminidase
3037
Multiple antibiotic resistance (MarC)-related protein
3038
42/62
Hyp. Prot.
3039
3040
53/68
3041
61/72
3042
51/69
Hyp. Prot.
3043
Colwellia psychrerythraea 34H (CPS)
Hyp. Prot.
1018
1019
51/68
Glucose-6-phosphate isomerase
1020
CPS_1021
34/50
Tryptophan halogenase, putative
1022
Tryptophan halogenase, putative
1023
Hyp. Prot.
1024
β-hexosaminidase
1025
ATPase
1026
1027
48/64
1028
40/59
Hyp. Prot.
1029
Shewanella denitrificans OS217 (Sden)
NADH dehydrogenase
2710
2709
48/65
Sden_2708
32/49
Tryptophan halogenase
2707
ATPase
2706
2705
45/58
2704
47/60
Hyp. Prot.
2703
2702
38/58
SapC
2701
F
Type II secretion system locus / xcs
xcsE
xcsD
xcsC
TonB-dependent receptor
Hyp. prot. / putative lipoprotein
Hyp. Prot.
Xanthomonas campestris pv. campestris str. AT 33913 (XCC)
3424
3425
3426
XCC3427
3428
3429
Sphingopyxis alaskensis RB2256 (Sala)
0316
58/71
0315
45/63
0314
35/46
Sala_0313
40/56
0312
46/58
Leu tRNA
Transcriptional regulator, XRE family
0311
Sphingomonas sp. (SKA58)
09626
63/74
09631
48/64
09636
32/47
2OG-Fe(II) oxygenase
09641
Hyp. Prot.
09646
SKA58_09651
39/56
09656
42/56
TPR repeat protein
09661
Caulobacter crescentus CB15 (CC)
0174
65/78
0173
55/70
0172
31/48
CC0171
39/55
0170
38/54
Hyp. Prot.
0169
Maricaulis maris MCS10 (Mmar10)
0382
57/72
0381
39/57
0380
30/40
Mmar10_0379
36/55
0378
39/55
Ornithine decarboxylase
0377
G
Hyp. Prot.
TonB-dependent receptor
Hyp. Prot. / Putative ABC transporter
Hyp. Prot. / δ-1-pyrroline-5-carboxylate reductase
ABC transporter ATP-binding protein
Hyp. Prot.
Transcriptional regulator
Xanthomonas campestris pv. campestris str. AT 33913 (XCC)
3713
XCC3714
3715
3716
3717
3718
3719
Caulobacter crescentus CB15 (CC)
Penicillin-binding protein, 1A family
1516
CC1517
39/54
1518
59/69
1519
54/68
1520
52/66
Antibiotic acyltransferase
1521
Saccharophagus degradans 2-40 (Sde)
Methionine biosynthesis MetW
3612
Sde_3611
37/55
3610
51/65
3609
36/58
3608
38/55
Pilus retraction protein PilT
3607
Pseudoalteromonas atlantica T6c (Patl)
Peptidase M24
Patl3777
Patl_3776
37/54
3775
36/53
3774
36/56
3773
49/63
Phytanoyl-CoA dioxygenase
3772
Sphingomonas sp. (SKA58)
ATPase
03850
03845
54/65
SKA58_03840
37/53
03835
66/75
03830
59/72
03825
59/72
Hyp. Prot.
03820
